# DKK1 Mediated Inhibition of Wnt Signaling in Postnatal Mice Leads to Loss of TEC Progenitors and Thymic Degeneration

**DOI:** 10.1371/journal.pone.0009062

**Published:** 2010-02-08

**Authors:** Masako Osada, Logan Jardine, Ruth Misir, Thomas Andl, Sarah E. Millar, Mark Pezzano

**Affiliations:** 1 Department of Biology, The City College of New York, New York, New York, United States of America; 2 Vanderbilt University Medical Center, Nashville, Tennessee, United States of America; 3 Departments of Dermatology and Cell and Developmental Biology, University of Pennsylvania, Philadelphia, Pennsylvania, United States of America; New York University, United States of America

## Abstract

**Background:**

Thymic epithelial cell (TEC) microenvironments are essential for the recruitment of T cell precursors from the bone marrow, as well as the subsequent expansion and selection of thymocytes resulting in a mature self-tolerant T cell repertoire. The molecular mechanisms, which control both the initial development and subsequent maintenance of these critical microenvironments, are poorly defined. Wnt signaling has been shown to be important to the development of several epithelial tissues and organs. Regulation of Wnt signaling has also been shown to impact both early thymocyte and thymic epithelial development. However, early blocks in thymic organogenesis or death of the mice have prevented analysis of a role of canonical Wnt signaling in the maintenance of TECs in the postnatal thymus.

**Methodology/Principal Findings:**

Here we demonstrate that tetracycline-regulated expression of the canonical Wnt inhibitor DKK1 in TECs localized in both the cortex and medulla of adult mice, results in rapid thymic degeneration characterized by a loss of ΔNP63^+^ Foxn1^+^ and Aire^+^ TECs, loss of K5K8DP TECs thought to represent or contain an immature TEC progenitor, decreased TEC proliferation and the development of cystic structures, similar to an aged thymus. Removal of DKK1 from DKK1-involuted mice results in full recovery, suggesting that canonical Wnt signaling is required for the differentiation or proliferation of TEC populations needed for maintenance of properly organized adult thymic epithelial microenvironments.

**Conclusions/Significance:**

Taken together, the results of this study demonstrate that canonical Wnt signaling within TECs is required for the maintenance of epithelial microenvironments in the postnatal thymus, possibly through effects on TEC progenitor/stem cell populations. Downstream targets of Wnt signaling, which are responsible for maintenance of these TEC progenitors may provide useful targets for therapies aimed at counteracting age associated thymic involution or the premature thymic degeneration associated with cancer therapy and bone marrow transplants.

## Introduction

The thymus serves two functions essential for a properly functioning adaptive immune response. These are the generation of new T cells from hematopoietic stem cells (HSC) and the selection of T cells expressing a functional self-tolerant T cell receptor (TCR) repertoire. These critical processes are controlled by the unique epithelial microenvironments found in the thymic stroma [1]. The stroma is broadly divided into two distinct regions, called the cortex and the medulla, containing epithelial cells that are functionally and phenotypically distinct. Epithelial cells in the thymic cortex are responsible for the attraction of T cell precursors, commitment to the T cell lineage, expansion of immature double negative (DN) thymocytes and positive selection of double positive (DP) thymocytes [2]. The proper formation of this key thymic microenvironment is dependent on interactions between developing thymocytes and thymic epithelial cells called thymic crosstalk [3], [4]. Mesenchymal cells are also required for the initial development and subsequent maintenance of a functional thymic microenvironment [5], [6]. The thymic medulla is composed of a heterogeneous population of epithelial cells that provide a microenvironment for newly positively selected CD4 and CD8 single positive (SP) thymocytes. Proper organization and development of mature mTECs requires Rank and CD40 mediated crosstalk from Lymphoid Tissue inducer cells (LTi) [7] and mature SP thymocytes [8], [9], [10], [11], [12] as well as γ/δ T cells [13]. Medullary thymic epithelial cells (mTECs), acting together with MHC class II^+^ dendritic cells, function to negatively select thymocytes that bear high affinity self-reactive TCRs [14]. MTECs also express a wide array of tissue restricted antigens (TRAs), so called “promiscuous gene expression” [15], [16], [17], some of which appear to be under the control of the AIRE transcription factor [18]. These TRAs represent a pool of self-antigens, which are used to negatively select auto-reactive thymocytes to induce self-tolerance or differentiation of regulatory T cell subsets. In addition to their critical role in tolerance induction, mTECs may also regulate post selection differentiation events including up-regulation of early T cell activation markers, as well as expansion of SP thymocytes prior to their export from the thymus [19]. The complexity of the thymic structure together with the need for cell-to-cell interactions in both the development and maintenance of the TEC microenvironments has hindered efforts to identify the molecular signaling pathways required for TEC development and function *in vivo*.

The Wnts represent a highly conserved family of 19 lipid-modified secreted glycoproteins in humans (18 in mice), thought to be critical to the development and maintenance of several organ systems [20], [21]. Wnt signaling regulates cell fate, establishment of the dorsal axis, asymmetric cell division, progenitor-cell proliferation and survival during embryonic, as well as postnatal development [22]. Appropriate regulation of Wnt signaling has also been shown to be important in the maintenance of hematopoietic [21] and epithelial stem cells [23], [24]. There are both canonical and non-canonical signaling pathways, driven by different combinations of the Wnt/Frizzled (Fz) complex. The most studied canonical Wnt pathway leads to stabilization of β-catenin by inactivating the “destruction complex” consisting of adenomatous polyposis coli (APC), Axin and glycogen synthase kinase 3β (GSK3β). In the absence of Wnt signaling, casein-kinase1α (CK1α)) and GSK3β phosphorylate β-catenin, which leads to the degradation of β-catenin. Binding of Wnt to its receptor Fz and co-receptor, low-density lipoprotein receptor related protein (LRP), releases Axin from the destruction complex, allowing amino-terminally dephosphorylated β-catenin to accumulate in the cytoplasm. This stabilized β-catenin then translocates to the nucleus where it engages with the lymphoid enhancer factor (LEF)-1, as well as the T-cell factors (TCF)-1, TCF-3 or TCF-4. The binding of β-catenin to LEF or TCF initiates transcription of genes such as *axin*, *cyclin D1* and *c-MYC* (for review see [25]). However, in most cases the specific targets of Wnt signaling remain elusive. Wnt receptor binding is highly regulated through association with diverse secreted proteins including Wnt inhibitory factor (Wif)-1, soluble frizzled (sFz), Dickkopf (Dkk) [26], Frzb-1 or Cerberus [27], as well as Kremen1 and 2 [28]. Wif-1 and sFz compete with Fz by binding available Wnts.

There are four Dickkopf-related protein (Dkk) family genes in the human genome, and three in that of the mouse. Dkk family members (DKK1 to DKK4) are secreted proteins with two cysteine-rich domains, separated by a linker region. DKK1, DKK2, and DKK4 function as antagonists of canonical Wnt signaling by binding to LRP5/6, preventing LRP5/6 interaction with Wnt-Frizzled complexes. DKK1, 2, and 4 also bind cell surface Kremen1 or 2 and promote the internalization of LRP5/6. Antagonistic activity of DKK3 has not been demonstrated [29]. DKK proteins have distinct patterns of expression in adult and embryonic tissues and have a wide range of effects on tissue development and morphogenesis. DKK1 is involved in a variety of craniofacial developmental processes and behaves as a strong head inducer and limb regulator [30]. DKK1 knockout mice are embryonic-lethal as they lack anterior head structures [31]. The expression of DKK1 is associated with sites of programmed cell death during limb development, while the loss of DKK1 expression results in the fusion of digits and formation of ectopic digits similar to mice with mutations in other proteins that regulate programmed cell death in the limb [31], [32], [33]. However, a direct relationship between DKK1 inhibition of the Wnt/β-catenin pathway and the induction of apoptosis has not been demonstrated. Transgenic keratin 14 (K14)-driven DKK1 over-expression blocked hair follicle development, as well as tooth and mammary gland development before the bud stage [34]. Tetracycline-inducible expression of DKK1, in lung epithelium, resulted in disruption of distal airway development and expansion of proximal airways through an N-myc, BMP-4 and FGF dependent mechanism [35]. Ectopic expression of DKK1 in K5-expressing epithelium, blocked taste papilla development as well as innervation of the tongue [36]. Recently, the same inducible transgenic model was used to inhibit Wnt signaling in wounded skin, resulting in a complete block in the development of new hair follicles derived from resident epithelial stem cells, while ectopic expression of Wnt7a driven by the K14 promoter lead to a 50% increase in the number of hair follicles that developed [37]. Taken together these studies demonstrate the importance of canonical Wnt signaling in regulating development of a number of epithelial tissues and organs as well as validating the use of ectopic Dkk1 expression to inhibit Wnt signaling.

We and others have demonstrated that both thymocytes and TECs express Wnt proteins, as well as their receptors and associated regulatory molecules and that TEC lines and primary TECs are capable of responding *in vitro* to Wnt proteins [38], [39], [40], [41]. A key role for the Wnt signaling cascade in controlling thymocyte cellularity and differentiation is apparent from studies using Tcf-1/LEF-1 knockout mice, as well as a number of complementary gain-of-function and loss-of-function studies [42], [43], [44], [45], [46]. Wnts secreted by both TECs and thymocytes were shown to regulate *Foxn1* expression, which is the transcription factor critical for thymic epithelial development and responsible for the athymic nude phenotype when mutated in mice and humans [38]. Deletion of APC, mediated by a K14-cre transgene, resulted in severe defects in thymic architecture marked by an increase in immature TECs expressing both K14 and K8. However, premature death of these mice, due to loss of APC in other epithelia, prevented analysis of the role of Wnt signaling in TEC maintenance in adult mice [47]. Evidence from our earlier characterization of Kremen1 KO mice, which exhibit increased canonical Wnt signaling in TECs, due to the lack of Wnt inhibitor Kremen1, suggests that canonical Wnt signaling may contribute to the maintenance of a less differentiated K5K8DP TEC progenitor population [39]. This is not surprising, as Wnt signaling is known to be important in progenitor/stem cell self-renewal [48]. These data were difficult to attribute solely to increased Wnt signaling in TECs, as the general loss of Kremen1 expression also affects lymphoid and mesenchymal cells in the thymus. Most recently, expression of a stabilized form of β-catenin, directed to TECs by a *Foxn1* promoter, blocks the initial commitment of endodermal epithelia to a thymic fate, subsequently interfering with thymocyte recruitment and preventing normal thymic function [49]. These results clearly demonstrate that precise regulation of Wnt signaling within the epithelial primordium is critical for normal TEC development and function, however, the early block in thymic development observed in this system precluded study of the role of canonical Wnt signaling in TEC maintenance in adult mice.

In this study we examined the effect of inhibiting Wnt signaling in TECs by crossing mice expressing the potent canonical Wnt inhibitor DKK1, controlled by a tetracycline-responsive regulatory element (*TetO-DKK1*) [50] with mice harboring a K5 promoter-driven tet inducer-VP16 transgene (*K5rt*TA) [51]. The resulting double transgenic *tetO-DKK1*;*K5rtTA* (tetO-Dkk1) mice produce high levels of DKK1 in both cortical and medullary TEC subsets, in the presence of doxycycline, leading to inhibition of canonical Wnt signaling [35], [36], [37], [50]. The advantage of the use of this transgenic model is that it allowed direct assessment of the contribution of canonical Wnt signaling to the maintenance of adult thymic epithelial architecture without affecting the initial development and organization of the thymus or other epithelial organs requiring Wnt signaling during fetal development. The effect of increasing Wnt signaling was also examined in a transgenic mouse expressing Wnt7a driven by a human K14 promoter [37] as well as fetal thymic organ cultures treated with Wnt3a conditioned medium. Since i*n situ* hybridization revealed more abundant DKK1 expression in the cortex, including the K5K8DP TEC subset thought to contain a TEC progenitor population, we reasoned that driving expression of the potent canonical Wnt signaling inhibitor DKK1, would have a profound effect on TEC development if Wnt signaling or its proper regulation were important in the maintenance of the postnatal TEC architecture. Inhibition of canonical Wnt signaling in adult mice through Tet-driven DKK1 expression resulted in a reduction in the number of all TEC subsets, a loss of K5K8DP TECs and a dramatic premature thymic degeneration.

## Methods

### Mice

Dr. Adam Glick (Penn State University) generously provided *K5rt*TA mice. *TetO-Dkk1 K5rtTA* and *K14-Wnt7a* mice have been described previously [37], [50], [51], as well as their use to inhibit Wnt signaling during epithelial development in other organs and tissues [36], [37], [50], [52]. *C57BL/6J* mice were obtained from the Jackson Laboratory (Bar Harbor, ME). *K5rtTA* mice were bred to *tetO-Dkk1* mice to generate *tetO-Dkk1*;*K5rt*TA double transgenic (DT) animals for study. Adult *tetO-Dkk1*;*K5rt*TA DT and littermate heterozygous control animals were fed mouse diet containing doxycycline (Dox) (2 g/kg, BioServe, NJ) for 4–8 weeks beginning at 6 weeks-of-age, unless otherwise indicated. For recovery experiments mice were fed Dox for 4 weeks and then food was changed to normal mouse chow for 2–4 weeks to allow recovery of thymic architecture. All mice were bred and maintained at the City College of New York animal facility and all experiments were performed with approval from the City College of New York institutional animal care and use committee.

### Antibodies

The following primary antibodies were used for experiments: anti CD45-PE Cy7 (clone 30-F11, BD Biosciences), I-A/I-E-PE (clone M5/114.15.2, BD biosciences), I-A/I-E-FITC (clone M5/114.15.2, eBioscience), I-A/I-E-APC (clone M5/114.15.2, eBioscience), Ulex europaeus agglutinin-1 (UEA1)-Biotin (Vector), UEA1-PE (Vector), cytokeratin 5 (MK-5, Covance), cytokeratin 14 (MK-14, Covance), Troma I (Developmental Studies Hybridoma Bank, IA), Troma III (Developmental Studies Hybridoma Bank, IA), MTS10 (kindly provided by Dr. Richard Boyd from Monash University, Australia), DNp63 (clone N-16, Santa Cruz), Aire (clone M-300, Santa Cruz), EpCAM-PerCP (clone G8.8, Santa Cruz), Ki67-FITC (clone B56, BD Biosciences), Ki67 (clone SP6, LabVision), CD4-PE Cy7(clone RM4-5, BD Biosciences), CD8-PerCp Cy5.5 (clone 53–6.7, BD bioscience), CD25-PE (clone PC6I, BD Biosciences) and CD44-APC (clone IM7, BD Biosciences). The following secondary reagents were used for experiments: donkey anti rabbit IgG-TRITC, donkey anti rabbit IgG-Cy5, donkey anti rat IgG-TRITC, donkey anti goat IgG-FITC, goat anti rat IgM-TRITC (Jackson ImmnoResearch), donkey anti rabbit IgG-FITC (Santa Cruz), anti rat IgG2a-FITC, anti rat IgM-FITC, stretavidin-APC, stretavidin-APC Cy7 (BD Biosciences) and stretavidin-TRITC (Southern Biotechnology Associate).

### Thymic Stromal Cell Preparation

Embryonic thymi were digested with collagenase D (1.2 mg/ml, Roche Diagnostics), DNase I (1.5 mg/ml, Roche Diagnostics), and Dispase (1.25 mg/ml, Invitrogen) at 37°C for 15 minutes with occasional gentle agitation with a glass pasture pipette. The resulting single cell suspension was washed with PBS and passed through 100 µm strainer (BD Biosciences) to remove any remaining undigested tissue. Adult thymi were cut into small pieces and majority of thymocytes were released by gentle agitation using a glass pasture pipette. The resulting tissue fragments were digested with collagenase D (1.2 mg/ml, Roche Diagnostics) and DNase I (1.5 mg/ml, Roche Diagnostics) for 15 minutes at 37°C followed by collagenase D (1.2 mg/ml, Roche Diagnostics), DNase I (1.5 µg/ml, Roche Diagnostics) and Dispase (1.25 mg/ml, Invitrogen) for 5 minutes at 37°C with occasional agitation using a glass pasture pipette. The single cell suspension was washed with PBS and passed through a 100 µm strainer (BD Biosciences).

### DKK1 In Situ Hybridization and K5 K8 Immunofluorescence Staining to Demonstrate Transgene Expression

*In situ* hybridization to detect DKK1 expression was performed as previously described [53]. Briefly, TetO-Dkk1 double transgenic mice and ST littermate control animals were fed Dox for 5 days prior to tissue harvest to allow DKK1 transgene expression while minimizing DKK induced thymic architecture defects. 12 µm frozen sections were prepared, air dried and fixed in 4% paraformaldehyde. *In situ* hybridization was then performed using anti-sense and sense probe templates for *Dkk1* synthesized by PCR of E14.5 mouse cDNA, using primers containing T7 RNA polymerase binding sites to amplify *Dkk1*: NM_010051. Following hybridization with DIG-labeled probes, bound probe was detected with anti-DIG Alkaline Phosphatase (AP) conjugate (Roche) followed by NBT/BCIP AP substrate (Roche). Following development of *in situ* signal, sections were counterstained with anti-K5 and anti-K8 antibodies followed by fluorochrome conjugated secondary reagents to allow co-localization of *in situ* signal with particular TEC subsets.

### Flow Cytometry

Cells were suspended in 100 µl of FACS staining buffer (FSB-1% fetal bovine serum, 5 mM EDTA and 0.02% NaN_3_ in PBS) with appropriately diluted primary antibodies for 20 minutes on ice in the dark. Secondary antibodies appropriately diluted in FSB were added cells were incubated for an additional 20 minutes on ice in the dark. After washing, cells were resuspended in 500 µl of FSB for data acquisition. Intracellular staining for Ki67 was performed using the FoxP3 staining kit (eBioscience) according to the manufacturer's instructions. TUNEL assays were performed using the *In Situ* Cell Death detection kit (Roche Diagnostics) according to the manufacturer's instructions. Live/dead discrimination was applied using ToPro3 (Invitrogen). Data acquisition was performed using an LSRII analyzer complete with three lasers (BD Biosciences) and cell sorting was performed using a FACS Aria (BD Biosciences). FACS data was analyzed using Flow Jo software (Tree Star).

### Immunohistochemistry and Confocal Microscopy

Fresh tissues were embedded in OCT medium (Fisher); snap frozen and sectioned (8 µm) using a Leica CM1950 Cryostat. Sections were air dried on bond-rite slides and then fixed in 4% paraformaldehyde or 100% ice-cold acetone. Sections were washed with PBS and blocked with blocking buffer (1% BSA, 0.1%Triton-X, 5% normal serum in PBS) for 10 min. Sections were incubated with appropriately diluted primary antibodies in blocking buffer in a humidified chamber for 1 hour at 37°C followed by incubation with secondary reagents diluted in blocking buffer in humidified chamber for 30 minutes at 37°C, then mounted with ProLong gold anti-fade reagent with DAPI (Invitrogen). Isotype control staining was performed for all primary antibodies to ensure specificity of staining. Images were acquired using Zeiss LSM510 confocal microscope and analyzed using LSM software (Zeiss).

### Quantification of Foxn1 Protein Expression In Vivo Using Confocal Microscopy

Frozen sections of thymic tissue were prepared and stained as described above. Confocal images were prepared by scanning each fluorochrome independently with a 40X lens with an optical slice thickness of 1.1 µm using a Zeiss LSM510 microscope. Cortical and medullary areas were defined by staining with anti-DEC205 and anti-K14, respectively, as well as TEC morphology in transitional zones. Thymic sections derived from control ST littermate and TetO-Dkk1 mice were placed on the same slide and stained with the same diluted antibody mix. Comparative analysis was always performed on sections in the same relative position on the slide and using identical laser power and detector voltage settings to ensure differences in Foxn1 fluorescence intensity were reflective of differences in protein expression. The Zeiss LSM software package was utilized to determine the Foxn1 fluorescence intensity of individual nuclei within either the cortex or medulla based on counterstaining. A total of 85 individual cells were scanned for each region of the thymus in both ST control and TetO-Dkk1 thymic tissues, following a 4-week induction of DKK1 with Dox. Tissues were derived from 3 independent experiments. Mean Foxn1 MFIs were determined for each condition and region. A students T test was performed to determine the significance of differences in Foxn1 fluorescence intensities calculated for each strain and TEC subset.

### Quantification of the Relative Area of K5K8DP TECs as well as the Numbers of ΔNp63^+^ TECs and Aire^+^ TECs Using Confocal Microscopy

Frozen sections of thymic tissue were prepared and stained as described above. Confocal images were prepared by scanning each fluorochrome independently with a 20X lens with an optical slice thickness of 2 µm using a Zeiss LSM510 microscope. The line scan tool provided in the LSM software (Zeiss) was used to scan several areas of sections that were not stained with the appropriate antibodies to determine the background thresholds used for the co-localization tool in the calculation of the relative area of K5K8DP TECs. A mean background plus 5% was used as the minimum threshold for each specific fluorochrome. After calculating the thresholds the co-localization tool was set to colorize all cells within the entire scan which co-expressed both K5 and K8 proteins blue. The scan frame was always identical for ST control and TetO-Dkk1 sections and scans were oriented to have the cortico-medullary junction bisect the scan. Comparative analysis was performed on control and experimental sections on the same slide and stained with the same antibody preparation. The relative area of each section which co-expressed both K5 and K8 was calculated using the Zeiss LSM image analysis software. The mean relative area and standard deviation were calculated using a minimum of 6 sections of each thymus taken from various regions within each thymic lobe and derived from 5 independent experiments.

For calculations of the number of Aire*^+^* and ΔNp63*^+^* TECs, sections were scanned as described above for Aire, ΔNp63 and UEA1. UEA1 and Aire staining were used to define the limits of the thymic medulla. An overlay box enclosing 10 mm^2^ of thymic area was then sequentially moved over the entire image and the number of Aire+ and ΔNp63*^+^* nuclei was counted within each 10 mm^2^ area for both the cortex and medulla. Sections were prepared from the central area of the thymus to ensure inclusion of both cortical and medullary areas. A minimum of 6 sections from each thymus derived from at least 5 independent calculations was used to calculate the mean and standard deviation of the number of Aire*^+^* and ΔNp63*^+^* TECs. DAPI staining was used to confirm that each fluorescent spot counted was indeed a nucleus, as partial nuclei were often encountered in the sections.

### RNA Isolation and Real-Time PCR

Total RNA was isolated from sorted thymic epithelial cells using Trizol reagent (Invitrogen). RT-PCR was performed using SuperScript III first-strand synthesis system (Invitrogen). Real-Time PCR was performed using the TaqMan gene expression assay system with primer and probe sets for DKK1 and GAPDH on a 7500 real-time PCR system (Applied Biosystem). Relative expression values for each sample were normalized against endogenous control GAPDH and the 2^−DDCt^ method was used to calculate the relative level of target mRNA.

### Fetal Thymic Organ Cultures

15.5 day-old fetal thymic lobes were dissected from C57BL/6 timed pregnant females, and incubated in 100 µl RPMI1640 complete media containing 10% fetal bovine serum supplemented with 100 µl Wnt3a conditioned medium harvested from L-cells transfected with a Wnt3a construct. Controls consisted of thymic lobes cultured in 100 µl of RPMI1640 complete medium supplemented with 100 µl of conditioned medium harvested from control non-transfected L-cells. FTOC cultures were performed in V-bottom 96-well plates (BD) in a high oxygen (70% O_2_, 25% N_2_, 5% C0_2_) chamber at 37°C. After 72 hrs, thymi were washed in 10% complete RPMI and lobes were embedded in OCT and snap frozen for sectioning.

### Statistical Analysis

Data comparison was performed using the Student's *t*-test function on the Excel software. A *P*-value of <0.01 was considered significant.

## Results

### Inhibition of Canonical Wnt Signaling with DKK1 in TECs Results in Rapid Thymic Degeneration in Adult Mice

**Figure 1:**
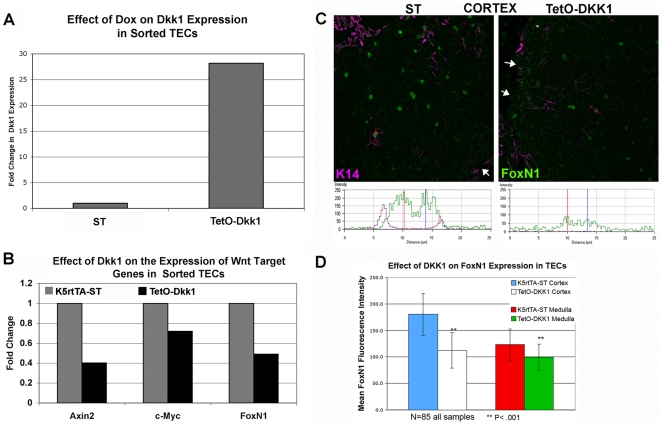
Relative expression of Dkk1, Wnt target genes and Foxn1 in sorted TECs (CD45^−^ MHCII^+^EpCAM^+^) as well as TECs *in vivo*. 4-week old tetO-Dkk1;*K5rt*TA and tetO-Dkk1-ST littermate mice were fed Dox food for 4 weeks prior to harvest and enzymatic dissociation of thymic tissue. Viable CD45^−^MHCII^+^EpCAM^+^ TECs were sorted to >98%purity by FACS. Subsequent to mRNA isolation, real-time PCR was performed using primer and probe sets specific to Dkk1, Axin2, c-Myc, Foxn1 and GAPDH. Relative Dkk1, Axin2, c-Myc and Foxn1 expression were normalized against endogenous GAPDH expression. (**A**) Relative expression of the Dkk1 transgene following 4-week Dox induction in TECs. (**B**) Relative expression of the Wnt target genes Axin2, c-Myc and Foxn1 in sorted TECs following 4-week Dox induction of the Wnt inhibitor Dkk1. (C) Representative 400X confocal scans of the thymic cortex of ST (left) and TetO-Dkk1 (right) thymus following a 4-week Dox feeding to demonstrate the reduced Foxn1 protein expression resulting from DKK1 transgene induction. Sections were stained with K14 (pink) Foxn1 (green) and DEC205 (not shown for clarity of Foxn1 analysis). Red arrow in each scan shows the position and direction of a line scan used to generate the quantitative analysis of Foxn1 protein expression shown in the histograms below each image. White arrows show the edge of the thymic section. (**D**) Histogram comparing mean Foxn1 fluorescence intensity in cTECs vs. mTECs. The mean Foxn1 fluorescence intensity was calculated from both cTECs and mTECs derived from 4-week Dox treated ST or TetO-Dkk1 mice (based on their location within DEC205+ and K14+ regions of the thymus). Means for each location and strain were calculated from a total of 85 individual cells from 3 independent experiments. Error bars in the histograms represent standard deviation. **Statistical significance was determined using a T test and P<.001.

Several previous studies have demonstrated a role for canonical Wnt signaling in regulating early T cell development [46], [54], as well as the development of the thymic epithelium [38], [39], [47], [49] however, due to early death of the mice or early blocks in thymic development, analysis of a role for Wnt signaling in the maintenance of adult TEC microenvironments was not possible. In an effort to specifically address the contribution of canonical Wnt signaling in the maintenance of postnatal thymic epithelial microenvironments, mice containing the potent canonical Wnt inhibitor DKK1, controlled by a tetracycline-responsive regulatory element (*TetO-Dkk1*) [53] were crossed with mice harboring a K5 promoter-driven tet inducer-VP16 transgene (*K5rt*TA) [51]. We hypothesized that the resulting double transgenic *tetO-Dkk1*;*K5rt*TA mice would produce high levels of DKK1 in the K5-expressing TEC subsets in response to the Dox, including the dominant K5+ mTEC subset and the K5K8DP cTECs. To demonstrate that the Tet-inducible double transgenic system resulted in increased expression of the canonical Wnt signaling inhibitor within TECs, *tetO-Dkk1*;*K5rt*TA (TetO-Dkk1) and *tetO-Dkk1-;K5rtTA*+ single transgenic (ST) littermate mice were fed Dox for 4 weeks beginning at 6-weeks-of-age. Real-time PCR performed on FACS sorted TECs (CD45^−^MHCII^+^EpCAM^+^) revealed a dramatic 25 fold increase in DKK1 expression within the TECs in the TetO-Dkk1 mice, when compared to TECs derived from littermate ST controls (Figure 1A).

**Figure 2:**
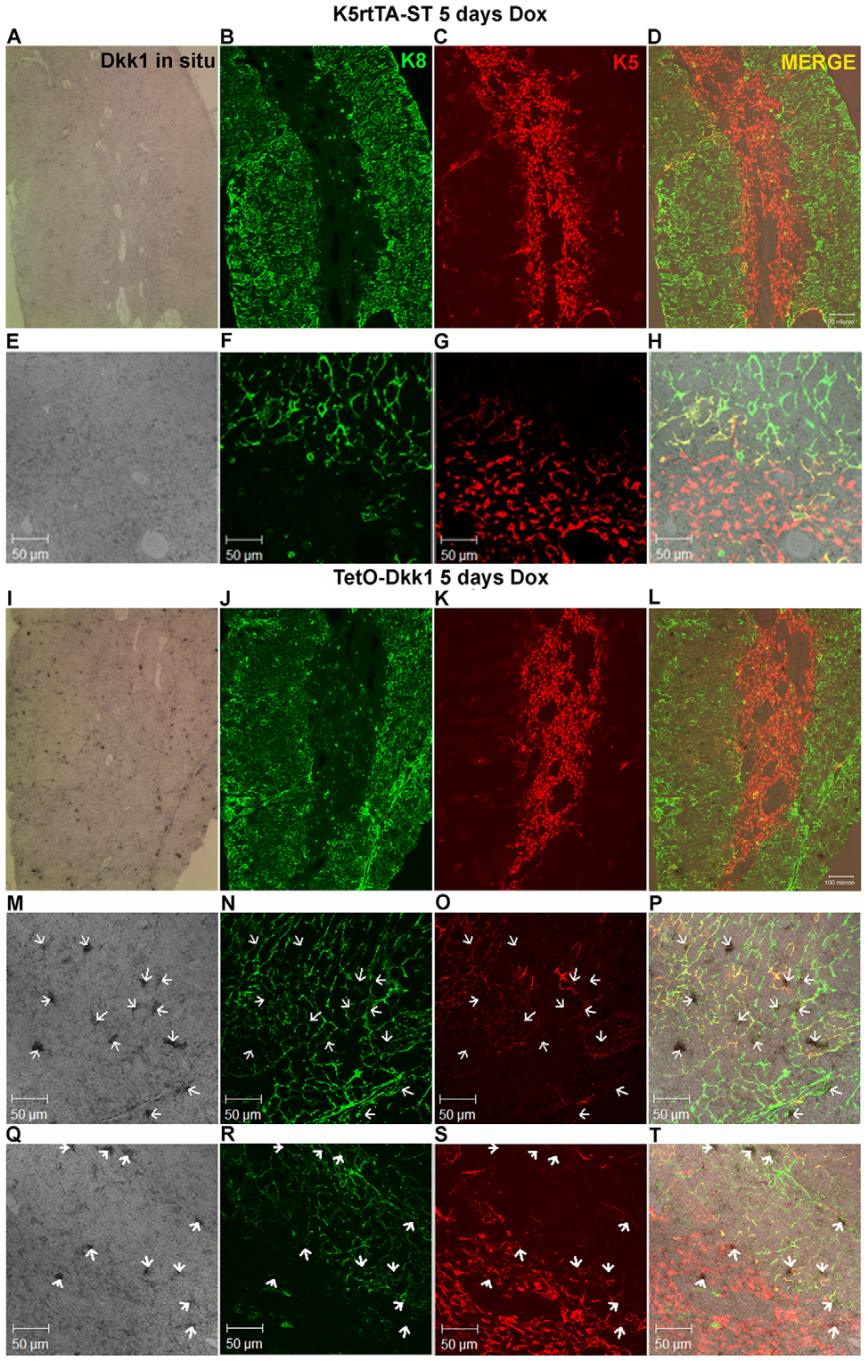
Doxycycline-regulated expression of the DKK1 transgene is evident in both cTECs and mTECs in the adult thymus. Following a 4-week Dox induction, 12 µm frozen thymic sections were prepared from ST control and TetO-Dkk1 mice and subjected to *in situ* hybridization with a DKK1 specific probe followed by detection with Sections were then counterstained with anti-Keratin 8 and anti-keratin 5 antibodies to allow localization the DKK1 expression to specific thymic regions and to cortical and medullary TEC subsets. Each row of photos shows *in situ* hybridization with a DKK1 specific probe followed by Immunofluorescent staining with K8 (green), K5 (red) and a merge of all three. K5rtTA-ST control lobes show little endogenous DKK1 expression at 100X (**A–D**) or 400X (**E–H**). In contrast, Dox induction resulted in widespread transgenic expression of DKK1 in the TetO-Dkk1 mice with positive TECs being more abundant in the K8 dominated cortex than the K5 dominated medulla when examined at 100X (**I–L**). High magnification examination of the thymic cortex of TetO-Dkk1 mice revealed that a high percentage of K5K8DP and a smaller number of K8+ cTECs exhibited strong DKK1 transgene expression. White arrows in all panels identify DKK1-expressing TECs, thus allowing characterization of the keratin profile (**M–P**). Surprisingly, more limited transgenic DKK1 expression was evident in the medulla, with a small percentage of the K5-expressing mTECs and a higher frequency of the K5K8DP TECs at the cortico-medullary junction expressing DKK1 (**Q–T**).

To determine the specificity and spatial orientation of transgenic DKK1 expression within the thymus, *in situ* hybridization with a DKK1 specific probe was performed on thymic sections derived from either control ST or TetO-Dkk1 mice following 5 days of Dox induction. Following *in situ* hybridization, immunofluorescence staining was also performed with anti-Keratin 8 and anti-Keratin 5 antibodies to allow localization of the *in situ* signal with particular TEC subsets. The short induction time was utilized to ensure that the assessment of DKK1 transgene expression was performed before significant changes in TEC subsets or thymic epithelial organization were apparent. *In situ* hybridization of thymic sections derived from ST mice with a DKK1 specific probe, revealed a very low level endogenous expression of DKK1, with no apparent pattern of distribution within the K8 dominated cortex or K5 dominated medulla when viewed at 100x (Figure 2A–D) or 400x (Figure 2E–H). In contrast, *in situ* hybridization of thymic sections derived from TetO-Dkk1 mice revealed a distinct pattern of DKK1 transgene expression within both cortical and medullary areas of the thymus, with a greater abundance of DKK1 expression within the thymic cortex and cortico-medullary junction (Figure 2I–L). When viewed at higher magnification, it is clear that DKK1 transgene expression is restricted to keratin+ TECs; however, DKK1 expression is not restricted to K5-expressing cells. More abundant DKK1 expression was observed in the cortex within primarily K5K8DP cells however; expression was also detected within K8 expressing cTECs (Figure 2M–P). More limited expression of the DKK1 transgene was apparent within K5-expressing TECs in the medulla and within K5K8DP TECs at the cortico-medullary junction (Figure 2Q–T).

Confirmation that transgenic expression of the Wnt inhibitor DKK1 resulted in a decrease in canonical Wnt signaling within the TECs was evident from the 30–60% decrease in the expression of the Wnt target genes Axin2 and c-Myc observed in TECs sorted from TetO-Dkk1 mice, when compared with identical populations sorted from Dox treated ST controls (Figure 1B). Similar reductions in Foxn1 expression were also observed (Figure 1B), supporting previous reports of a role of Wnt signaling in regulating Foxn1 expression in TECs *in vitro* [38].

Immunofluorescent staining of thymic sections with Foxn1 antibody allowed assessment of the level of Foxn1 protein expression within distinct TEC subsets in response to a 4-week Dkk1-mediated inhibition of Wnt signaling. Counterstaining these sections with anti-DEC205 and anti-K14 to define cortical and medullary areas of the thymus, respectively, revealed a lower Foxn1 staining intensity in both cTECs (Figure 1C) and mTECs (Figure S1). Quantitative confocal analysis was performed utilizing the Zeiss LSM software to scan individual CTEC and mTEC nuclei to determine the mean fluorescence intensity of Foxn1 antibody staining in ST control and TetO-Dkk1 sections. Representative scans shown for cortical areas in figure 1C (red arrow represents scan), together with the histograms showing the reduced Foxn1 staining intensity in the Dox treated TetO-Dkk1 thymus. A total of 85 cTEC and mTEC nuclei were scanned for each strain from 3 independent pairs of ST and TetO-Dkk1 mice and the resulting mean fluorescence intensities of Foxn1 were graphed in figure 1D. A significant reduction (P<0.001) in mean Foxn1 fluorescence intensity was detected in both cTECs and mTECs, with the most pronounced decrease apparent in cTECs which changed from a mean of 181 (ST) to a mean of 112 (TetO-Dkk1) representing a 40% decrease in Foxn1 expression. MTECs showed a more modest but still highly significant 20% reduction in Foxn1 expression. This difference could reflect a differential effect of DKK1 on cTECs and mTECs or might reflect the higher level of DKK1 transgene expression observed in the cortex.

**Figure 3:**
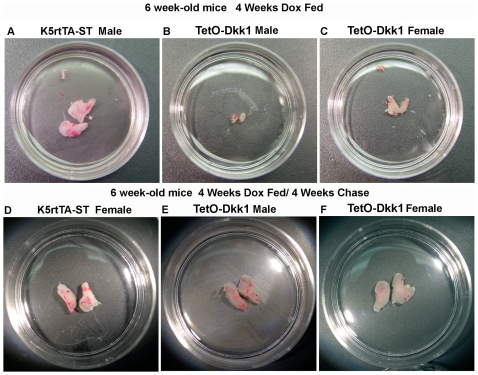
Effect of DKK1 expression on thymus size. Upper row shows photographs of intact thymic lobes to demonstrate the dramatic thymic involution observed after 4 weeks of doxycycline driven Dkk1 expression. (**A**) Control Male K5rtTA-ST, (**B**) Male TetO-Dkk1 and (**C**) Female TetO-Dkk1. Lower row demonstrates the recovery of thymic size of doxycycline treated lobes following a 4 weeks chase period. (**D**) Control Female K5rtTA-ST, (**E**) Male TetO-Dkk1, (**F**) Female TetO-Dkk1. Female K5rtTA-ST mice exhibited a similar thymic size to male controls after Dox feeding, as did Male K5rtTA-ST mice after 4 weeks Dox feeding and 4 weeks chase (data not shown).

Analysis of thymus size in response to Dox-driven DKK1 expression revealed a dramatic reduction in overall thymic size (Figure 3, *A–C*) with the most significant reduction in male mice (82% based on weight) while a 42% reduction was observed for females. This difference in size was representative of similar results obtained in 4 different experiments performed when Dox feeding was initiated at 6 weeks of age and carried out for a total of 4 weeks. Longer feeding of Dox, up to 8 weeks, resulted in almost a complete loss of the thymus, with the residual thymus remaining resembling a severely atrophied thymus that might be found in a mouse older than 1 year-of-age and making isolation of intact thymic tissue for analysis difficult.

**Figure 4:**
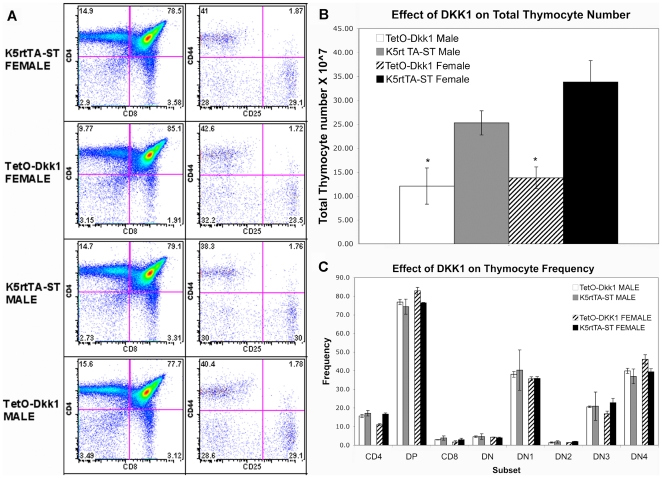
Effect of DKK1 on total thymocyte number and subset frequency. **A**. Thymocyte FACS profiles representative of 4 independent experiments showing the frequency of thymocyte subsets including the DN1-SP subsets. No apparent differences in profile between Dox fed TetO-Dkk1 male and female when compared with similarly treated K5rtTA littermate controls. **B**. Total thymocyte number is dramatically reduced in both male and female TetO-Dkk1 mice following 4 weeks of Dox feeding, when compared with Dox fed ST littermate controls. **C.** Following 4 weeks of Dox feeding, FACS analysis of thymocytes derived from either male or female TetO-Dkk1 mice showed no changes in the frequency of individual thymocyte subsets. (The frequency of individual DN subsets represents the frequency of the total number of CD4-CD8- cells) Means for each subset were determined from 4 independent experiments with at least 3 mice of each sex and genotype.

To examine if DKK1-mediated inhibition of canonical Wnt signaling in TECs, impacted T cell development, total thymocyte numbers were counted. In addition, FACS analysis of thymocytes derived from TetO-Dkk1 and K5rtTA-ST mice following 4 weeks of Dox feeding using antibodies against CD4, CD8, TCRβ, CD25 and CD44 was used to assess differences in thymocyte subset frequency. FACS profiles representative of 4 independent experiments are shown in Figure 4A. Total thymocyte numbers were reduced by more than 50% in both male and female TetO-Dkk1 mice when compared with identically treated K5rtTA-ST littermate controls (Figure 4B). FACS analysis of thymocytes revealed no significant differences in the frequency of any thymocyte subsets (Figure 4C), suggesting that expression of DKK1 in TECs does not lead to stage specific blocks in T cell development but rather a general loss of thymic capacity maintain sufficient numbers of T cells, possibly due to a loss of epithelial niches.

**Figure 5:**
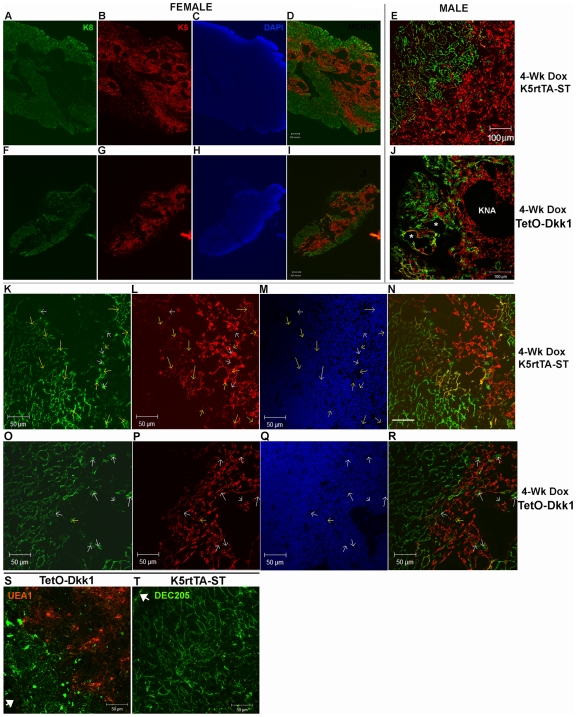
Effect of Dkk1 expression on the distribution of TEC subsets defined by K5 and K8 expression. Frozen thymic sections prepared from K5rtTA-ST and TetO-Dkk1 mice after 4 weeks of doxycycline feeding to induce DKK1 expression. Low magnification images of thymic sections derived from female K5rtTA-ST mice (**A–D**) and TetO-Dkk1 mice (**F–I**) stained with anti-K8 (green) to identify the cortex and anti-K5 (red) to identify the medulla as well as DAPI. Merged images of K5 and K8 staining (**D vs. I**) reveal K5K8DP TECs thought to contain progenitors (yellow). Similar images of merged K5 and K8 staining from male K5rtTA-ST (**E**) and TetO-Dkk1 mice (**J**) reveal a more dramatic loss of cortical architecture and altered cTEC morphology. * -Identify cystic structures and abundant keratin negative areas (KNA) in the TetO-Dkk1 mice. Higher magnification images of female K5rtTA-ST mice stained with K5 and K8 antibodies reveal abundant K5K8DP TEC progenitors at the CMJ as well as within the medulla (**K–N**, yellow arrows). (White arrows show K8SP thought to be mature mTECs) Similar sections derived from littermate TetO-Dkk1 mice reveal an absence of the K5K8DP TEC progenitors at both the CMJ and within the medulla (**O–R**). Further evidence of the hypoplastic cortex and loss of the reticular cTEC network is revealed when TetO-Dkk1 mice with severe phenotypes are stained the cTEC specific DEC205 antibody (green) and UEA1 (red) to define medullary areas. (**S**). Compared with similar staining of K5rtTA-ST littermate mice (T). A dramatic loss of cortical area and the typical reticular cTEC morphology is apparent in the TetO-Dkk1 thymus. White arrows show the outer edge of the thymus.

To determine the effect of DKK1-mediated inhibition of canonical Wnt signaling in TECs, on TEC development and organization, histological analysis of thymic sections derived from adult Dox treated TetO-Dkk1 mice and ST control mice, was performed with a panel of antibodies used to define thymic architecture. K8 is expressed in the majority of cTECs and a subset of mature mTECs, while K5 expression is found predominantly in immature mTEC subset as well as more rare cells scattered in the cortex. Cells that co-express both K5 and K8 are thought to contain a subset of TEC or cTEC progenitors [55], [56] and are primarily localized to the cortico-medullary junction (CMJ) with additional cells scattered through the medulla. Comparative analysis of female DKK1 transgenic mice stained with K8 and K5 antibodies at low magnification revealed a dramatic loss of both cortical and medullary TECs, with a pronounced thinning of the K8 expressing cTECs (Figure 5, *F–I*) when compared with Dox fed ST littermates (Figure 5, *A–D*). Both the control K5rtTA-ST and TetO-Dkk1 mice show abundant keratin negative areas in the medulla, which is a characteristic of CD1 background mice not normally found in inbred strains like C57BL/6. Similar K8 and K5 histological analysis of sections derived from Dox-treated male TetO-Dkk1 mice revealed almost a complete loss of normal cTEC organization and an abundance of aberrant cystic structures (Figure 5, J *), while identically treated ST male littermate mice showed a normal distribution and morphology of both cTECs and mTECs (Figure 5, *E*). Examination of thymic sections derived from Dox-treated female mice at higher magnification revealed almost a complete loss of K5K8DP TEC progenitors in TetO-Dkk1 mice, including cells at both the CMJ and within the medulla (Figure 5, *O–R*). Yellow arrows in identical positions within the individual color panels allow identification of K5K8DP TECs. The remaining K8SP TECs in the medulla of DKK1 mice (see white arrows) were primarily the mature UEA1 bright cells (data not shown). Comparative analysis of sections derived from Dox-treated K5rtTA-ST mice revealed abundant K5K8DP TEC progenitors at the CMJ as well as within the medulla (yellow arrows, Figure 5, *K–N*). Male mice showed similar reductions in K5K8DP TECs but more severe reductions in cTEC and mTECs and a more significant disruption of cTEC morphology.

Staining with the cTEC specific DEC205 antibody revealed a severely hypoplastic cortex in Dox-treated TetO-Dkk1 mice. This is apparent from the reduced cortical area evidenced by the close proximity of the medulla, defined by the red UEA1^+^ mTECs, to the edge of the thymus (white arrow, Figure 5S). The cTECs also appear to have lost the normal reticular morphology and more expansive cortex, which are apparent in the control Dox-treated ST mice (Figure 5T). At 400× magnification, no UEA1^+^ medulla was visible when photographs were taken at the outer cortex of control mice. The reduced cortical area and loss of typical reticular morphology is similar to the phenotype described for the involuted thymus of aged mice [57], [58]. Low magnification DEC205 staining of TetO-DKK1 and K5rtTA-ST controls is provided in supplemental Figure 1 similarly demonstrating the reduced cortical area and disrupted cTEC organization in response to DKK1 expression.

**Figure 6:**
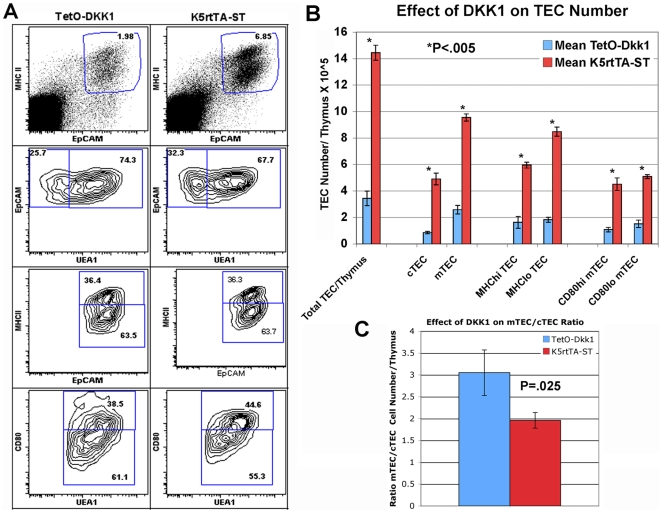
FACS Analysis of TEC profile and quantification of TEC numbers in response to DKK1 mediated inhibition of Wnt signaling. Following a 4-week Dox induction of DKK1 expression, thymic lobes from TetO-Dkk1 and K5rtTA-ST littermate mice were dissociated using Collagenase/Dispase/DNase digestion. CD45 magnetic beads were used to partially deplete CD45^+^ hematopoietic cells from the resulting single cell suspension, prior to staining with antibodies against CD45, MHCII, EpCAM, and CD80 together with UEA1 lectin. TetO-Dkk1 mice showed a dramatic decrease in the frequency of EpCAM^+^ MHCII^+^ CD45^−^ TECs (**A**, upper left) when compared with K5rtTA-ST controls (**A**, upper right panel). Further analysis of the CD45-EpCAM^+^ TECs using the MHCII and EpCAM to distinguish the MHCII^lo^ immature and MHC^hi^ mature TEC subsets, revealed no differences in the frequency of these subsets (**A**, third set of panels) Similarly, separation of the UEA1^+^ mTECs into UEA1^+^CD80^hi^ mature and UEA1^+^CD80^lo/neg^ immature mTEC subsets, revealed no affect of DKK1 induction on the frequency of mTEC subsets (A, lower set of panels). (**B**) Comparison of the mean total CD45^−^MHCII^+^EpCAM^+^ TEC numbers and the mean number of each TEC subset including EpCAM^+^UEA1^−^ cTECs, EpCAM^+^ UEA1^+^ mTECs, EpCAM^+^MHC^hi^ mature TECs, EpCAM^+^MHC^lo^ Immature TECs, UEA1^+^CD80^lo^ immature mTECs and UEA1^+^CD80^hi^ mature mTECs, following 4-weeks Dox feeding. Means represent the total cell number/thymus, calculated from 3 independent experiments utilizing 5-pooled dissociated thymi from each strain. Error bars show standard deviation. * P<.005 demonstrating significant reductions in TEC number for all subsets analyzed (**C**) Dox treatment of TetO-Dkk1 mice results in an increase in the mean mTEC/cTEC ratio (blue bar) when compared to identically treated K5rtTA-ST mice (red bar). Means calculated based on 3 independent experiments as described above. Error bars =  standard deviation. P = .025.

In an effort to quantify the effect of transgenic expression of DKK1 on the frequency of total TECs and specific TEC subsets, thymic lobes from TetO-Dkk1 and K5rtTA-ST littermate mice were dissociated using Collagenase/Dispase/DNase digestion following 4 weeks of Dox feeding. A total of 5 thymi from each strain were pooled for each experiment to yield sufficient numbers of TECs for analysis and 3 independent experiments were performed. CD45 magnetic beads were then used to partially deplete CD45^+^ hematopoietic cells from the resulting single cell suspension, prior to staining with antibodies against CD45, MHCII, EpCAM, and CD80 together with UEA1 lectin. CD45^−^ MHCII+ EpCAM^+^ cells are defined as TECs, while immature and mature TECs within this population are defined as MHC^low^ EpCAM^+^ and MHC^hi^EpCAM^+^ subsets, respectively. After gating on TECs further analysis with EpCAM versus UEA1 allowed separation of EpCAM^+^ UEA1^+^ mTECs and EpCAM^+^ UEA1^−^ cTECs. Analysis of the mTEC population with anti-CD80 allowed separation of the mature CD80hi and immature CD80 lo/neg mTEC subsets. A representative FACS TEC profile derived from 4-week Dox fed female TetO-Dkk1 and littermate ST mice is shown in Figure 6A. A total cell count, following CD45 depletion, was used together with the TEC subset frequencies to analyze the effect of DKK1 on total TEC and TEC subset numbers/thymus (Figure 6B). Following Dox feeding, TetO-Dkk1 mice showed a dramatic decrease in the frequency of CD45^−^ EpCAM^+^ MHCII^+^ TECs (mean **2.48+/−0.68%,** representative freq. Figure 6A, upper left panel) when compared with K5rtTA-ST controls (**8.3+/−1.94**%, upper right panel) suggesting a loss of TECs within the smaller thymus. To confirm that this difference in TEC frequency was representative of an actual reduction in TEC numbers, the frequencies of TECs in each subset were used to calculate and compare absolute TEC numbers. Analysis of total TEC numbers revealed a significant decease (P<.005) in the number of TECs present in Dox treated TetO-Dkk1 mice (mean = 3.43+/− 0.35×10^5^/thymus) when compared to littermate ST controls (mean = 14.44+/−0.56×10^5^/thymus). Similar significant reductions in the number of all other TEC subsets analyzed was apparent in the Dox-treated TetO-Dkk1 mice, including cTECs, mTECs, total immature MHC^lo^TECs, total mature MHC^hi^TECs, as well as both mature CD80^hi^UEA1^+^ and immature CD80^lo/neg^UEA1^+^ mTECs (Figure 6B). While no other significant differences in the frequency of the various TEC subsets defined by differences in the expression of MHCII, UEA1 and CD80 were apparent, analysis of the CD45^−^EpCAM^+^ TECs using the mTEC specific lectin UEA1 revealed a small but repeatable reduction in the frequency of EpCAM^+^ UEA1^−^ cTECs in the Dox treated TetO-Dkk1 mice (23.3+/−2.12%) when compared with ST controls (31.4+/−5.3%) (Figure 6A, second set of panels). When the absolute numbers of cTECs and mTECs in Dox-treated TetO-Dkk1 mice were compared with identically treated ST littermate controls the ratio of mTEC to cTECs showed a moderately significantly decreased (P = .025) from 3.05+/− 0.52 in TetO-Dkk1 mice to 1.96+/−0.18 in ST controls (Figure 6C). A repeatable but not significant reduction in the ratio of immature EpCAM^+^MHC^lo^ to mature EpCAM^+^MHC^hi^ TECs was also observed in Dox-fed TetO-Dkk1 mice. Taken together these results suggest that K5 promoter-driven DKK1 expression in postnatal TECs leads to a dramatically reduced frequency and absolute number of TEC characterized by significantly reduced numbers of all TEC subsets with a slightly more significant reduction apparent in the cTECs. Given the higher expression of the DKK1 transgene within the cortex and specifically within the K5K8DP TEC subset, as well as the loss of K5K8DP TECs observed in Dox-fed TetO-Dkk1 mice in histology, we wanted to quantify the loss of K5K8DP TECs.

**Figure 7:**
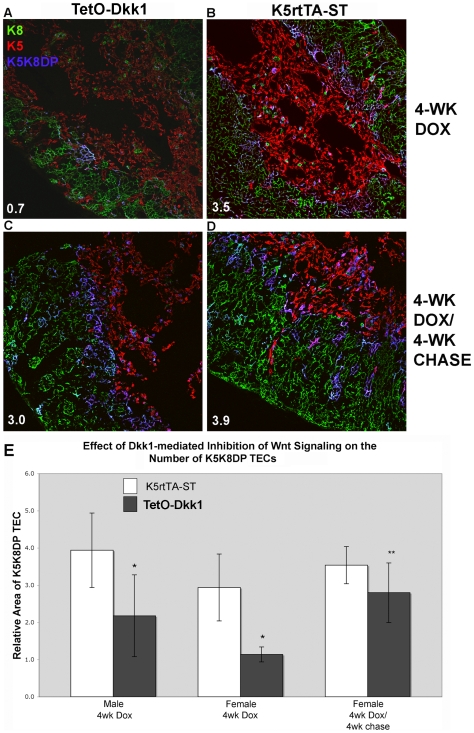
Inhibition of Wnt signaling leads to a decline in the number of K5K8DP TECs thought to contain a TEC progenitor population. A dramatic reduction in the abundance of K5K8DP TECs is apparent in confocal images of thymic sections derived from TetO-Dkk1 (**A**) when compared with K5rtTA-ST littermate animals (**B**) following 4 weeks of doxycycline feeding. These K5K8DP TECs reappear in 4-wk Dox treated TetO-Dkk1 mice following a 4 weeks chase of doxycycline withdrawal (**C**) possessing a similar frequency of K5K8DP TECs to that of similarly treated K5rtTA-ST mice (**D**). TECs, which exhibited fluorescence intensities 5% above the mean fluorescent background for K5 and K8 and co-expressing both keratins, are colored blue using the Zeiss LSM image analysis software. The relative percentage of TECs co-expressing K5 and K8 is shown by the white numbers in the lower left corner of each image. (Magnification = 200×) (**E**) The mean relative area of K5K8DP TECs in male and female K5rtTA-ST mice (White bars) and TetO-Dkk1 mice (Gray bars) following 4 weeks of Dox feeding and in female mice following a 4 weeks Dox chase experiment. Error bars show standard deviation. * - P value <.005. ** - P value no longer significant following recovery period. Means were calculated based on 5 independent experiments, which analyzed a minimum of 6 sections cut from various locations within the thymus of each strain (TetO-Dkk1 versus ST littermate) and each sex (N = 30).

Co-localization analysis of confocal images was utilized to quantify the decline in K5K8DP TEC, thought to contain or represent TEC progenitors, evident following 4 weeks of DKK1-mediated inhibition of canonical Wnt signaling. Male and female TetO-Dkk1 mice exhibited a 40% and 60% reduction in the mean relative area of K5K8 co-localization, respectively, when compared with littermate ST controls (Figure 7E, left two pairs of bars). These relative K5K8 co-localization values were means calculated based on the analysis of a minimum of 6 sections of each thymus derived from 5 independent experiments (N = 30). Representative sections used for analysis are shown in Figure 7A–B. The relative area of TECs exhibiting K5 and K8 co-localization in confocal images (blue colored cells) is shown in the lower left corner of each panel. This relative area was determined by setting an independent threshold for K5 and K8 staining at 5% above background fluorescence intensity. The Zeiss LSM image analysis software then colorizes all pixels, which exhibit fluorescence intensities above that threshold for both the K5 and K8 channels blue to indicate co-localization. The relative area of K5K8DP pixels is determined as a percentage of the total thymic area in the image. Note that the TECS co-expressing K5 and K8 and colorized blue that are affected by DKK1 expression, include a dominant population at the cortico-medullary junction, as well as a fairly large number of TEC within the medulla as well. Taken together these histological analyses suggest that canonical Wnt signaling is required to maintain the K5K8DP TEC subset thought to contain a TEC progenitor population or to control its development from an as yet undefined TEC stem cell population and that inhibition of Wnt signaling with DKK1 results in a premature thymic involution resulting in dramatic cortical defects.

### The Number of ΔNp63^+^ TECs Is Reduced following Tet-Regulated DKK1 Induction

**Figure 8:**
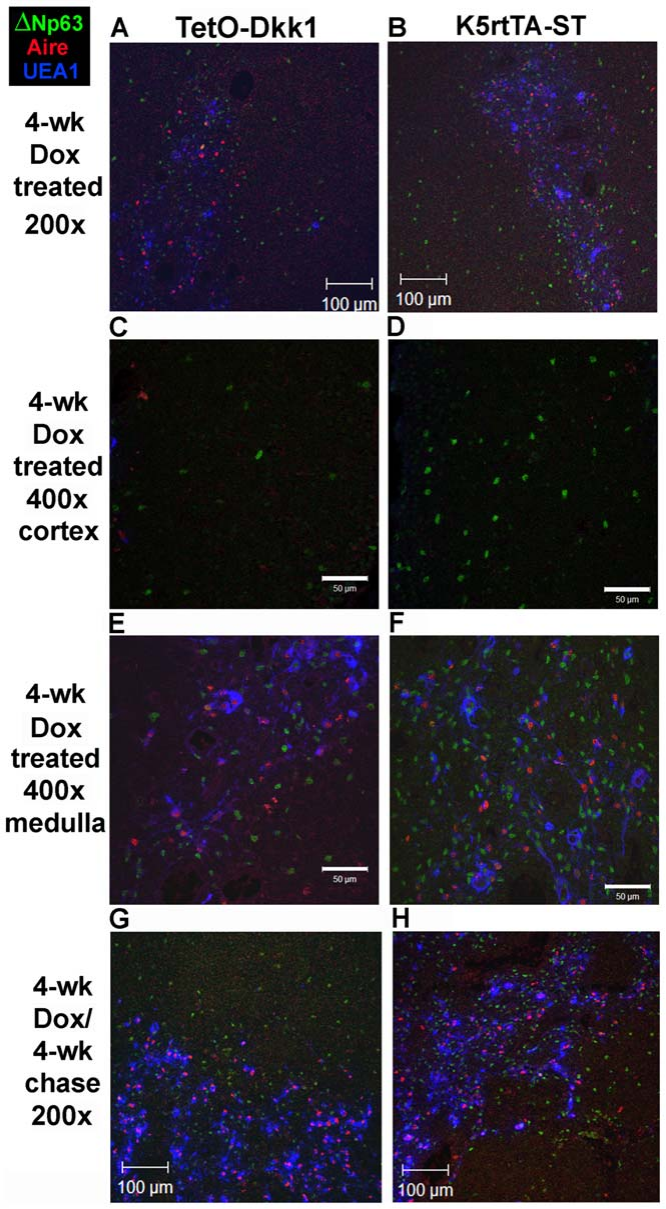
DKK1 Expression results in reduced ΔNp63^+^ and Aire^+^ TECs. Thymic sections derived from female TetO-Dkk1 (left column) and K5rtTA-ST mice (right column) stained with Anti-ΔNp63 (green), Anti-Aire (red) and UEA-1 (blue). **A–E** show thymic sections derived following 4 weeks of doxycycline feeding. Low magnification images of a large area of thymus from TetO-Dkk1 mice (**A**) compared with the K5rtTA-ST (**B**). Higher magnification images of the cortex (**C & D**) and medulla (**E & F**) in TetO-Dkk1 and K5rtTA-ST, respectively. Low magnification images of 4 week Dox treated thymic tissue derived from TetO-Dkk1 (**G**) or K5rtTA-ST animals following a 4-week recovery period.

**Figure 9:**
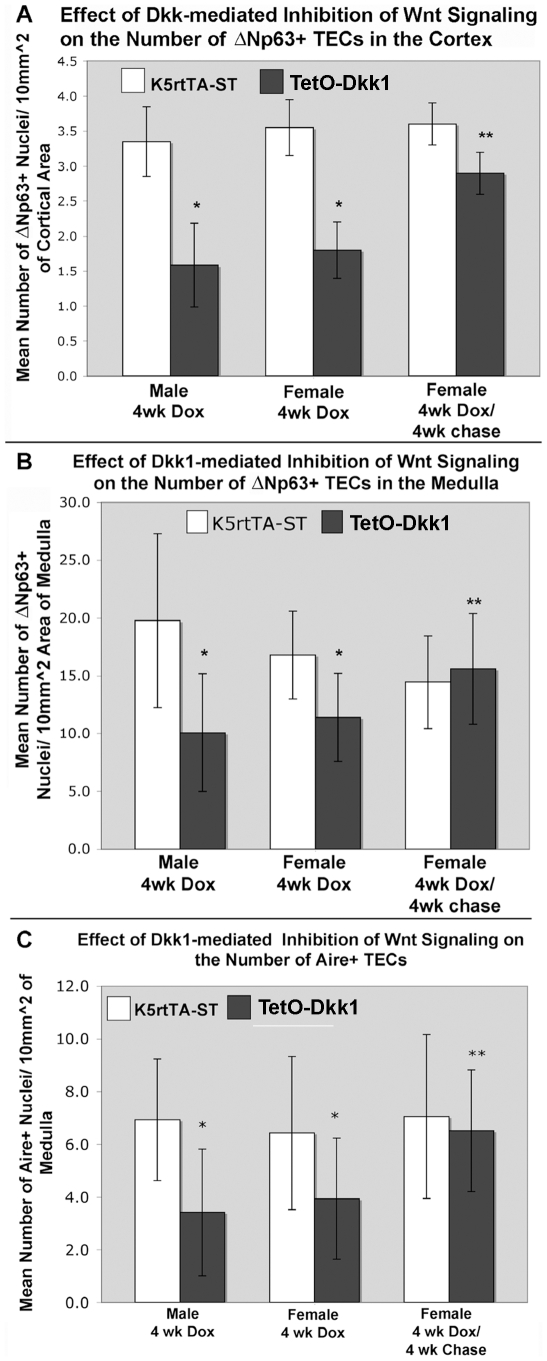
Effect of inhibition of Wnt signaling in the adult thymus on the number of ΔNp63^+^ and Aire ^+^TECs. (**A**) The mean number of ΔNp63**^+^** nuclei/10 mm^2^ area of cortex, calculated for K5rtTA-ST (white bars) and TetO-Dkk1 (gray bars) following 4 weeks of Dox feeding and after a 4 weeks Dox chase. (**B**) The mean number of ΔNp63**^+^** nuclei/10 mm^2^ area of medulla, calculated for K5rtTA-ST (white bars) and TetO-Dkk1 (gray bars) following 4 weeks of Dox feeding and after a 4 weeks Dox chase. (**C**) The mean number of Aire**^+^** nuclei/10 mm^2^ area of medulla, calculated for K5rtTA-ST (white bars) and TetO-Dkk1 (gray bars) following 4 weeks of Dox feeding and after a 4 weeks Dox chase. *-P value <.01, **- no longer significant.

The P53 family member, P63, is required for proper development of thymic stroma and appears to influence epithelial stem cells, or TECs early in ontogeny as P63 KO mice exhibit a hypoplastic non-functional thymus, lacking in mature TEC subsets [59], [60]. In stratified epithelium, the N-terminal truncated isoform ΔNp63 is an early marker of epithelial fate and is expressed in stem cells in the basal layer, as well as the proliferating transit amplifying progeny, but is lost in more mature epithelial populations [60], [61], [62], [63], [64], [65]. While epithelial ontogeny in the thymus is less clearly defined, Aire and CD80 expression appear to be restricted to more mature mTEC subsets. Ontogeny of cTECs remains undefined, although evidence from fetal mice suggests that either bipotent TEC progenitors or cTEC progenitors are found within the K5K8DP TEC subset. In the thymus ΔNp63 expression is extremely abundant, however similar to stratified epithelium ΔNp63 is rarely co-expressed with Aire in the mature mTEC subset (see Figure 8F and [66]), suggesting that at least with respect to mTEC ontogeny, ΔNp63 expression follows a similar pattern in the thymus with expression dominating immature subsets. Since DKK1 inhibition of Wnt signaling appears to affect TEC subsets defined by K5 and K8 co-expression, the effect of tet-induced DKK1 expression on the number of potentially immature ΔNp63^+^ TECs, as well as the frequency of mature Aire^+^ mTECs was examined using Immunofluorescent staining of thymic sections. Low magnification images derived from Dox-treated TetO-Dkk1 mice revealed loss of ΔNp63^+^ TECs throughout the thymus (Figure 8A) when compared with Dox-treated ST controls (Figure 8B). Higher magnification images of distinct thymic functional microenvironments clearly reveal both a reduced number of ΔNp63^+^ TECs and a reduced expression level within both the cortex (Figure 8C) and the medulla (Figure 8E) of TetO-Dkk1 animals when compared with ST controls (Figure 8D, cortex & F, medulla). The frequency of mature mTECs defined by Aire expression (red nuclei) was also reduced in Dox-treated TetO-Dkk1 mice when compared to control ST animals. To quantify the changes in both ΔNp63^+^ immature and Aire^+^ mature TECs resulting from DKK1 inhibition of Wnt signaling, the number of TECs expressing ΔNp63 and Aire were counted in 10 mm^2^ areas within confocal images prepared from either Dox-treated TetO-Dkk1 or ST control animals. Means were calculated from 4 independent experiments utilizing 3 animals from each strain and sex. The number of Aire+ or ΔNp63+ TECs was counted in a minimum of 50 (10 mm^2^) areas in sections derived from multiple thymic lobes for each mean. In male TetO-Dkk1 mice, the mean number of ΔNp63^+^ in the cortex was significantly reduced by >50% to 1.6 (+/−0.6) cells/10 mm^2^, when compared with ST controls containing 3.4 (+/−0.5) ΔNp63^+^ cTECs/10 mm^2^. Female TetO-Dkk1 mice showed a similar 50% reduction in the frequency of ΔNp63^+^ cTECs after Dox treatment (Figure 9A). The medulla of the thymus contains a higher density of ΔNp63^+^ TECs. Dox-treated TetO-Dkk1 female mice exhibited a 33% reduction in the mean number of ΔNp63^+^+ mTECs (11.4+/−3.8 cells/10 mm^2^) when compared to ST controls (16.8+/−5.1 cells/10 mm^2^) (Figure 9B middle bars). Male TetO-Dkk1 mice exhibited an even more significant 49% reduction in the frequency of ΔNp63^+^mTECs from 19.8+/−2.5 in ST controls to 10.1+/−5.0 in TetO-Dkk1 animals (Figure 9B, left bars). This more significant effect of inhibition of Wnt signaling on the number of ΔNp63+ TECs in male mice might contribute to the more pronounced thymic degeneration observed in male mice in response to DKK1 (see Figure 3 and 5). Similar ∼50% reductions in the frequency of mature Aire^+^ mTECs were also observed in TetO-Dkk1 mice when compared with ST controls in response to Dox feeding (Figure 9C). Again, the difference in the frequency of mature mTECs in males was slightly greater than that observed in females.

Both immature and mature TEC subsets appear to be reduced after Dox-mediated DKK1 expression, suggesting that Wnt signaling may lead to reduced proliferation of TECs or death to TECs at an immature stage. Proper regulation of Wnt signaling has been shown to be necessary for maintenance of the regenerative capacity of stem cell populations as well as stem cell to TA cell transition in other epithelial tissues [23], [24], [48], [66], [67], [68]. Whether a similar stem cell mechanism is responsible for initial development and maintenance of adult TECs is yet to be determined.

### DKK1 Induced Thymic Atrophy Is Reversible

To investigate whether progenitor cells in the thymus were permanently lost or simply blocked in development when Wnt signaling was inhibited, TetO-Dkk1 mice were treated with Dox for 4 weeks and then Dox food was withdrawn and replaced with normal mouse chow for a 4 week chase. The thymus size returned to normal in both male and female TetO-Dkk1 animals after a 4-week recovery period, when compared to similarly treated ST controls (Figure 3, lower row). This increase in total thymic size was accompanied by an expansion of the K5K8DP TEC subset at both the cortico-medullary junction and within the medulla (Figure 7C). A similar recovery of TECs was observed in male mice (data not shown). Quantitative analysis of TetO-Dkk1 mice showed that following a 4-week recovery period, the mean relative area of K5K8DP TECs recovered from the post-treatment 1.1+/− 0.2% to 2.8+/− 0.8%, which was no longer significantly different than identically treated ST controls, which contained a mean of 3.5+/−1.2% (Figure 7E). The frequency of both ΔNp63^+^ TECs (Figure 9, A&B) and mature Aire^+^ mTECs (Figure 9C) also returned to levels comparable with ST controls or non-Dox fed TetO-Dkk1 mice (data not shown). Representative fluorescent micrographs used for the quantitative analysis of TEC numbers, following the 4 weeks recovery period, are shown in Figure 8, G and H. The number of ΔNp63^+^ and Aire^+^ TECs clearly increases dramatically compared with TetO-Dkk1 sections obtained following 4 weeks of DKK1 mediated inhibition of Wnt signaling. Expression of the Wnt signaling inhibitor DKK1 in TECs localized primarily in the cortex leads to a rapid involution of the adult thymus. This involution is characterized by the loss of K5K8DP TECs as well as the loss of both ΔNp63^+^ and Aire^+^ TECs. This involuted thymus maintains the capacity to recover from the inhibition of Wnt signaling, as the thymus returns to a normal size with a normal distribution of TECs following inactivation of DKK1 expression (Figure 3D–F & Figure 7C–E).

### DKK1 Expression Leads to a Reduction in Proliferation of Immature TECs but Has No Effect on Apoptosis

**Figure 10:**
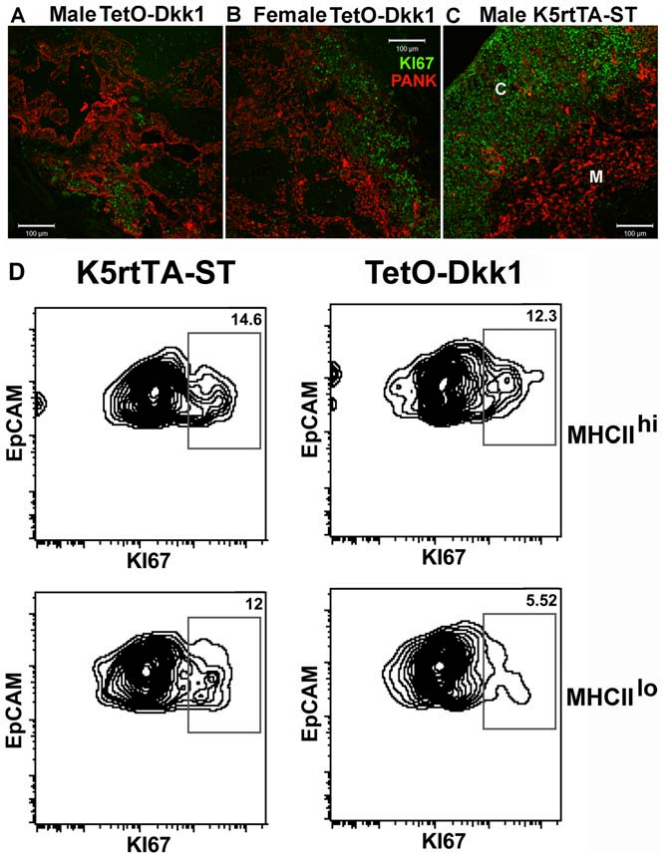
Inhibition of canonical Wnt signaling through transgenic expression of DKK1 leads to reduced proliferation of TECs. Sections of thymus derived from (**A**) Male,TetO-Dkk1 (**B**) Female TetO-Dkk1 and (**C**) Male K5rtTA-ST littermate control animals stained with anti-Pan-keratin antibody (red) and anti-Ki67 antibody after 4 weeks of Dox feeding. FACS analysis of dissociated thymic tissue derived from similarly treated female TETO-DKK1 and control ST mice revealed only a slight reduction in Ki67**^+^** cells within the mature CD45^−^ MHCII^hi^ EpCAM^+^ TEC subset in TetO-Dkk1 mice (**D**, upper panels). In contrast, a greater than 50% reduction in the frequency of cycling Ki67^+^ TECs was observed in the immature CD45^−^ MHCII^low^ EpCAM^+^ TEC subset in TetO-Dkk1 mice, when compared with Dox-treated ST littermate controls (**D**, lower panels). Positive gates for Ki67 staining within each sample were determined using rabbit isotype control antibody. The FACS data presented in **D** is derived from 5-pooled mice of each strain and the results are representative of 3 independent experiments.

In an effort to understand the role of Wnt signaling in maintaining adult thymic epithelial microenvironments and to identify the mechanism responsible for the dramatic decrease in thymic size in response to transgenic expression of DKK1, both the number of cycling TECs and the frequency of apoptosis were examined. Immunofluorescent staining of thymic sections, derived from Dox-treated TetO-Dkk1 mice, revealed a significant reduction in total Ki67^+^ cells in both male and female animals (Figure 10A&B) with the most dramatic reduction in cycling cells and the most significant disruption in normal epithelial organization (detected with Pan-keratin staining), observed in male mice (Figure 10A). Normal TEC organization was apparent in identically Dox-treated ST control animals, which showed a typical pattern of abundant Ki67+ cells in the cortex (primarily thymocytes) and proportionally lower numbers of cycling cells in the medulla (Figure 10C). Due to the close association of TECs with the more abundant thymocytes and the complex three-dimensional organization of the thymic stroma, it is difficult ascertain whether the reduced frequency of cycling cells in the TetO-Dkk1 thymus observed by histology was limited to thymocytes or also included TECs.

To specifically examine the impact of Tet-mediated DKK1 expression on the proliferation of TECs, flow cytometry was used to analyze Ki67 expression in dissociated thymic tissue derived from female mice after 4 weeks of Dox feeding. Female mice were chosen over male since the phenotype was less severe, ensuring the presence of all TEC subsets for analysis. Following enzymatic dissociation and partial magnetic depletion of CD45^+^ cells for TEC enrichment, cells were stained with CD45 EpCAM, MHCII and Ki67. Cells were initially gated for CD45^−^EpCAM^+^ TECs followed by separation of mature MHCII^hi^EpCAM^+^ and immature MHCII^low^EpCAM^+^ subsets, as shown in Figure 6. Analysis of mature TECs derived from Dox-treated TetO-Dkk1 animals revealed only a slight but repeatable reduction in the frequency of Ki67^+^ cycling cells within CD45^−^MHCII^hi^EpCAM^+^ TECs, when compared with Dox-treated ST animals (Figure 10D upper panels). In contrast, analysis of Ki67 expression within the immature MHCII^low^ EpCAM^+^ subset of TECs revealed a greater than 50% reduction in the number of cycling TECs derived from TetO-Dkk1 mice. The FACS profiles provided in Figure 10D are representative of the results obtained in 3 independent experiments.

**Figure 11:**
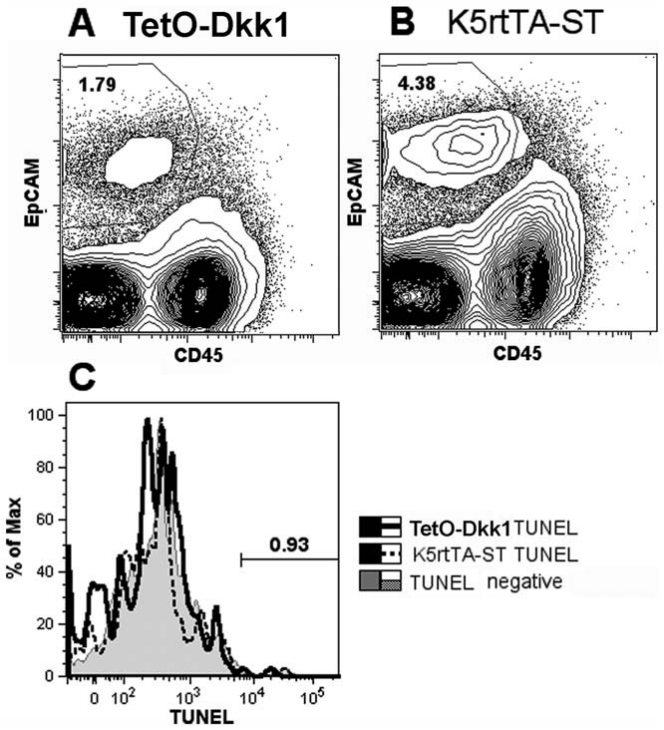
TEC TUNEL Assay: The effect of DKK1-mediated inhibition of canonical Wnt signaling on apoptosis in TECs. The gating strategy used to analyze total CD45^−^EpCAM^+^ TECs derived from TetO-Dkk1 female mice (**A**) and K5rtTA-ST littermate female control mice (**B**) after 4 weeks of Dox feeding to induce DKK1 expression. An overlay of the TUNEL staining for TECs from TetO-Dkk1 and K5rtTA-ST mice, as well as TetO-Dkk1 TECs, stained in the absence of TDT as a negative control (**C**), reveals no difference in apoptosis with the TECs.

Given the association between endogenous DKK1 expression and sites of apoptosis during limb development [31], [32], [33] it was reasonable to predict that the premature thymic involution observed in response to Tet-induced DKK1 expression in TECs could also result from an increase in apoptosis of TECs. To determine if inhibition of Wnt signaling by DKK1 was leading to increased apoptosis, a TUNEL assay was performed on dissociated thymic tissue derived from Dox-treated TetO-Dkk1 and ST mice after Dox treatment. No difference in the frequency of TUNEL^+^ cells was observed in the CD45^−^ EpCAM^+^ TEC subset (Figure 11). TUNEL and active caspase-3 staining of thymic sections counterstained to detect TECs confirmed that no difference in apoptosis of TECs in response to DKK1 expression was observed (data not shown).

Taken together these results suggest that inhibition of canonical Wnt signaling, through Tet-induced DKK1 expression within the majority of cTECs and a minor population of mTECs, results in a reduced number of cycling TECs, contributing to a decrease in frequency of K5K8DP putative TEC progenitors and premature involution of the thymus.

## Discussion

Previous studies have demonstrated a clear role of Wnt signaling and its soluble regulators in the development of thymocytes [41], [69], [70] as well as the initial development of the thymic epithelial cells [39], [47], [49] however, the lack of an inducible model and the subsequent early blocks or changes in development of the thymus seen in these systems, precluded analysis of the role of Wnt signaling in the maintenance of adult thymic epithelial microenvironments. In this study a transgenic model, which targeted expression of the potent canonical Wnt signaling inhibitor DKK1 to the majority of cTECs including the K5K8DP population at the CMJ and a minor population of mTECs (Figure 2), through the action of a tetracycline dependent regulatory protein expressed under the control of the K5-promoter, was used to demonstrate a requirement for canonical Wnt signaling in the maintenance of the adult thymus. Simply feeding the tetracycline analogue, doxycycline, to TetO-Dkk1 mice, allowed for conditional control of DKK1 expression, leading to a>25 fold increase in DKK1 mRNA levels within the TECs of adult mice following 4 weeks of Dox induction (Figure 1A). *In situ* hybridization of thymic sections, derived from Tet-ODkk1 mice fed Dox for 5 days, with DKK1 specific probe demonstrated that DKK1 expression was restricted to keratin + TECs with higher expression in the cortex than the medulla (Figure 2). The resulting inhibition of Wnt signaling within sorted TECs was confirmed by the expected decline in expression of known Wnt target genes including *Axin 2* and *c-Myc,* although a decline in Wnt target genes could also be explained by a loss of specific TEC subsets that differentially express these genes. Interestingly, a 50% reduction in the expression of *foxn1* was also detected (Figure 1B). Foxn1 is a key transcription factor required for proper epithelial morphogenesis and the capacity of thymic epithelial cells to attract lymphoid precursors from the bone marrow [72]. *Foxn1* expression was previously shown to be positively regulated by Wnt signaling in TECs [38], however in a recent study thymic epithelial development was blocked and *foxn1* expression was down-regulated in a transgenic mouse model that used the *foxn1* promoter to drive expression of stabilized β-catenin [49].

Expression of DKK1 in the postnatal thymus resulted in a dramatic premature involution of the thymus with male mice exhibiting the most significant decrease in thymic size compared with the reduction observed in females (Figure 3). This difference between the effects of DKK1 on male versus female mice might be attributed to the added burden of androgen receptor mediated involution observed in male mice [71], [72], [73]. In support of this idea, Kwack et al. showed that DKK1 expression was dramatically up regulated in response to dihydotestosterone in dermal papilla cells derived from balding skin. This increase in DKK1 lead to increased apoptosis *in vivo*, as well as decreased *in vitro* growth potential of keratinocytes [74]. The androgen driven increase in endogenous DKK1 expression in males together with the tet-mediated transgenic expression, may have contributed to the increased response observed in males. When male TetO-Dkk1 mice were surgically castrated prior to initiating Dox feeding, more modest reductions in thymic size comparable to TetO-Dkk1 females were observed (data not shown), supporting a synergy between androgen driven thymic involution and the degeneration induced by DKK1 expression in TECs.

The reduction of thymic size in TetO-Dkk1 mice following Dox treatment was associated with a greater than 50% decrease in the total number of thymocytes found in both male and female TetO-Dkk1 mice (Figure 4B). Analysis of thymocyte subsets by FACS revealed no significant differences in subset frequency (Figure 4A & C), suggesting that the inhibition of Wnt signaling in TECs does not lead to blocks in T cell development. Previous studies have demonstrated a requirement for Wnt signaling in T cell development at DN1 and DN3 and then again at DP stage [43], [44], [45], [46]. The decline in T cell number in TetO-Dkk1 mice appears to be the result of the loss in epithelial niches required to support thymocyte development, rather than a direct effect of DKK1 on the thymocytes themselves. The lack of an apparent T cell block also suggests that the defects in thymic architecture observed in TetO-Dkk1 mice are more likely the result of a direct effect of DKK1 on the TECs, rather than an indirect effect due to crosstalk. Regardless of the outcome of future studies aimed at understanding the differential effect of DKK1 on male versus female mice, the results of this study demonstrate for the first time a requirement for Wnt signaling in the maintenance of adult thymic epithelial microenvironments.

Histological analysis of thymic sections utilizing cortical and medullary specific markers following Tet-regulated DKK1 expression, revealed a hypoplastic cortex, marked by a loss of the normal reticular organization of cTECs (Figure 5) and an abundance of cystic structures (Figure 5J), however both cortical and medullary areas were reduced in size and were deficient in specific TEC subsets (Figures 6 & 7). Particularly apparent was a loss of K5K8DP cells at the cortico-medullary junction (CMJ) and scattered in the medulla (Figure 7). Age associated thymic involution is marked by a loss of a defined CMJ, a reduction in cortical area and an increase in epithelial free areas. Additionally, in humans there is an infiltration of the perivascular spaces by fat cells [57], [58], [75], [76]. Some aspects of the involuted thymic phenotype observed in TetO-Dkk1 mice following Dox-treatment resemble that observed in the aging thymus, including a reduced thymic size, reduced TEC and thymocyte cellularity, cortical degeneration and increased epithelial free areas. One distinction seems to be the decrease in K5K8DP TECs, thought to represent or contain a progenitor population [55], [56]. In contrast to the phenotype in TetO-Dkk1 mice, K5K8DP TECs have been reported to increase in the aging thymus [57], [58], [75]. The increase of K5K8DP cells in the aging thymus might be explained by the reported increase in senescent TECs with age [57] that then results in decreased proliferative potential of the immature TEC subsets, blocking their differentiation into more mature TECs. Alternatively, inhibition of canonical Wnt signaling may act on an undefined progenitor/stem cell population upstream of the K5K8DP subset in TEC ontogeny, although it is not clear whether the thymus is maintained by a stem cell mechanism or proliferation of more mature TEC subsets. Canonical Wnt signaling has been shown to be required for the initiation of hair follicle development from bulge stem cells [53], as well as *de novo* hair follicle regeneration during wound healing in skin, mediated by epithelial stem cells recruited from non-bulge areas. Ectopic Dkk1 expression completely blocked the development of these hair follicles as well as new stem cells within the hair follicle bulge [37]. Canonical Wnt signaling has also been shown to regulate *Foxn1* expression in TECs, the gene defective in nude mice [38]. Following Dox treatment sorted TECs exhibit a greater than 50% reduction in the level of *Foxn1* mRNA (Figure 1B) and a reduction in the both the frequency and intensity of Foxn1 staining in both cTECs and mTECs by histology (Figure 1C & D). A reduction in *Foxn1* mRNA expression could be explained by a loss of TEC subsets that express higher levels of *Foxn1*, given that *Foxn1* is differentially expressed within TEC subsets. However, the 40 & 20% reduction in Foxn1 protein expression observed in cTECs and mTECs, respectively, in response to transgenic DKK1 expression provides support for a direct effect of Wnt signaling on *Foxn1* expression. Loss of *Foxn1* results in aberrant epithelial morphogenesis and the inability of TECs to attract hematopoietic progenitors to the thymus. Recently, a very similar premature thymic degeneration phenotype observed in postnatal mice was linked to the dose of Foxn1 expressed by TECs resulting from expression of an altered Foxn1 allele [79]. Similar to the phenotype observed when Wnt signaling was inhibited by DKK1 older mice expressing the Foxn1^lacZ^ allele also exhibited reduced proliferation in the MHCII^lo^ TEC subset. The phenotype observed in response to DKK1-mediated inhibition of Wnt signaling may be the result of loss of Foxn1 expression contributing to a block in TEC development and/or altered TEC proliferation in progenitor subsets. Due to the high turnover of TECs recently reported [77], loss of the capacity of progenitor populations to maintain epithelial homeostasis then may contribute to the thymic degeneration observed, regardless of whether these progenitors represent true stem cells or simply less mature TEC subsets with the capacity to proliferate and maintain adult TEC microenvironments.

Cortical and medullary compartments of the thymus are typically defined by the expression of K8 and K14, respectively. Keratin 5 is often co-expressed with K14, however its distribution within the thymus is more extensive to include cells scattered throughout the cortex, as well as a more abundant population of K5K8DP TEC found at the cortico-medullary junction. Support for this population being a multi-potent progenitor is based on a similarity with fetal TECs, abundant in the E12 thymus, which have been shown to give rise to both cTECs and mTECs in clonal assays [78]. However, no direct lineage relationship has been demonstrated in the adult thymus. A strong circumstantial case has also been made to support the hypothesis that the K5K8DP population represents the immediate precursor to K8+ cTECs [55], [56]. The relationship between K5K8DP TECs and mTECs is less clear, primarily due to the widely accepted notion that the medulla is composed of a dominant K5^+^K8^−^ mTEC subset and a less abundant K8^+^K5^−^ mTEC subset thought to be the mature mTECs. Several recent studies and our own data presented here (Figures 5 & 7) have demonstrated that K8K14DP and K8K5DP TECs are more abundant in the medulla than previously thought [79], [80], [81] and might represent precursors to the mature K8SP mTECs.

The disappearance of the K5K8DP TEC population in response to DKK1-mediated inhibition of canonical Wnt signaling and their subsequent reappearance following inhibitor withdrawal demonstrate the requirement for Wnt signaling in the maintenance of K5K8DP TEC in both the cortex and medulla. Recovery following removal of Dox suggests that Wnt signaling either drives expansion of the K5K8DP population or regulates their differentiation from an undefined precursor cell. A role for Wnt signaling in the maintenance or expansion of the K5K8DP TEC population was supported by our previous study, where loss of the Wnt signaling inhibitor Kremen1 (a co-receptor for DKK1 with LRP) leads to increased Wnt signaling within TECs and an abundance of K5K8DP TECs together with cortical defects [39]. In another study, deletion of the APC gene in TECs, through expression of K14 driven Cre, resulted in a hypoplastic non-functional thymus with an abundance of K14K8DP TECs. These cells also exhibited increased β-catenin localization in the nucleus, a hallmark of canonical Wnt signaling [47]. In our own hands, Immunofluorescent staining of thymic sections derived from 6-week-old transgenic mice that express Wnt7a under the control of the human K14 promoter [37], known to drive transgene expression specifically in cTECs [82], [83] revealed an abundance of K5K8DP TECs scattered throughout the thymus including most of the cortex (Figure S2, A–D), while littermate control mice showed a normal distribution of K5^+^ TECs dominating the medulla with most K5K8DP TECs localized to the cortico-medullary junction (Figure S2, F-I). Visualization of the cortico-medullary junction at 400X revealed a less defined boundary and abundant K5K8DP TECs in the K14-Wnt7a mice with almost no K8 SP mature mTECs (Fig. S2E). In contrast, thymic sections from control mice had a defined cortico-medullary junction, fewer K5K8DP TECs and abundant K8SP mature mTECs (Fig. S2J, white arrows show K8SP mTECs). A similar abundance of K5K8DP TECs and loss of defined cortical and medullary areas were observed in thymic sections derived from E15.5 FTOCs following 72 hrs of culture in Wnt3a conditioned medium (Fig. S2K, upper row). Littermate E15.5 FTOCs cultured in FTOC medium in the absence of Wnt3a exhibited fewer K5K8DP TECs and more defined cortical and medullary areas (Fig. S2K, lower row). Most recently, Zuklys et al demonstrated that expression of stabilized β-catenin, under the control of the Foxn1 promoter, led to TECs following an altered epithelial fate or being halted very early in development with most of the TECs in the E13 thymus expressing both K5 and K8 [49], Unfortunately, the early blocks in thymus development Foxn1 promoter-driven stabilized β-catenin mice and the early demise of the conditional APC KO mice prevented analysis of adult TEC maintenance or comparisons with fetal development. However these studies, together with the data we present here showing that K5 promoter-driven DKK1 expression leads to thymic degeneration, identifies canonical Wnt signaling as an important target for future therapeutic strategies designed to counteract thymic involution.

Quantitative analysis of both mature Aire^+^ mTECs and cycling ΔNp63^+^ TECs in both the cortex and medulla showed that loss of Wnt signaling leads to a general loss of TECs with a more dramatic effect on immature ΔNp63^+^ cells (Figures 8 and 9) or an inhibition of p63 expression within TEC subsets. P63 deficient mice exhibit a severely hypoplastic non-functional thymus [59], [60] and appears to show the effect in early TECs development. P63 expression has been reported to identify epithelial stem cells [84] however, the abundance of p63-expressing cells in the thymus (Figure 8 & [79], [85], [86], [87], [88]) and other tissues like bladder epithelium indicates that p63 expression is maintained in the immediate progeny of the stem cells as well. In the skin, p63 expression is associated with K14^+^ progenitor cells in the basal layer and is lost as the cells lose proliferative potential and differentiate [62]. Differentiation is associated with expression of miRNA 203 which down-regulates p63 expression [89], [90]. The disappearance of ΔNp63^+^ TECs following DKK1 expression could then represent a loss of TEC subsets with proliferative potential. This could explain the involution of the thymus in TetO-Dkk1 mice following Dox induction of DKK1 expression. Alternatively, ΔNp63 has been shown to influence TEC development by regulating fibroblast growth factor receptor 2-IIIb (FgfR2-IIIb) and the Notch signaling component Jag2 [59], both of which have been shown to be critical to TEC proliferation or development.

Loss of ΔNp63^+^ cells in both the cortex and medulla coupled with the disappearance of the putative K5K8DP TEC progenitor population supports a common lineage or a common role for Wnt signaling in the maintenance or expansion of multiple TEC progenitors. Removal of DKK1 expression allowed for a full recovery of the thymus within 4 weeks including a return to normal thymic size (Figure 3), recovery of the cortex as well as the K5K8DP progenitor population (Figure 7), and a normal frequency of both immature and mature TEC subsets defined by ΔNp63 and Aire expression, respectively (Figure 8 and 9). The ability of the thymus to recover suggested that inhibition of Wnt signaling did not lead to death of the progenitor population but rather reduced cycling of either the progenitors themselves or their progeny. This was confirmed by the lack of TUNEL staining within the TECs (Figure 11), as well as decreased numbers of cycling Ki67^+^ TECs following DKK1 induction (Figure 10). The most pronounced decrease in cycling cells was observed in the MHCII^low^ EpCAM^low^ subset resembling the recently identified cTEC progenitors [91], and supporting a role for Wnt signaling in maintaining TEC progenitor/stem cell populations or promoting the development of their immediate progeny.

The decision, to remain a stem cell or to differentiate is thought to be controlled by competition for limited quantities of growth factors such as BMP, Hedgehog, FGF and Wnt within the stem cell niche, thereby maintaining a balance between stem cell self-renewal and differentiation [92]. Our results might suggest that the thymic degeneration observed in response to transgenic DKK1 expression results from a loss of TEC stem/progenitor cell maintenance or proliferation of an immature TEC subset. This idea is supported by a number of studies demonstrating the importance of Wnt signaling and Wnt proteins in the maintenance of stem cells of a variety of lineages. In the colon crypts, loss of Tcf4 leads to depletion of epithelial stem cells required for normal tissue homeostasis [24]. Inhibition of Wnt signaling through transgenic expression of DKK1 results in a complete loss of colon crypts in adult mice [66], [68]. *In vitro* stimulation of HSCs with Wnt3A leads to increased *Bcl2* (B-cell lymphoma 2) expression and increased self-renewal capacity, while inhibition of the canonical Wnt signaling through ectopic expression of Axin1 or with a truncated form of Frizzled resulted in decreased *in vitro* proliferation and *in vivo* repopulation capacity [48]. Wnt3A deficiency results in decreased numbers of HSCs in fetal liver and decreased self-renewal capacity [67]. Expression of constitutively active β-catenin in lymphoid or myeloid progenitors generated uncommitted cells with multilineage differentiation potential [93], suggesting that Wnt signaling has a role in maintaining an undifferentiated state. Most recently, analysis of mice engineered to express DKK1 in the osteoblastic HSC niche, indicated that the self-renewal of HSCs is negatively affected when Wnt signaling is inhibited by DKK1 [23].

The results of this study demonstrate for the first time that canonical Wnt signaling within TECs is required for the maintenance of epithelial microenvironments in the postnatal thymus. Loss of Wnt signaling within TECs results in a decrease in the K5K8DP subset localized at the cortico-medullary junction and a decline in the number of cycling TECs primarily within the immature subsets. Loss in TEC cycling then contributes to rapid thymic degeneration characterized by the loss of both TECs and developing thymocytes dependent on TEC niches for survival. The ability of the thymus to recover from this degeneration, following removal of the Wnt signaling inhibitor DKK1, suggests that Wnt signaling may be required for the expansion of the subset of TEC progenitors found within the K5K8DP population or their development from and as yet unidentified progenitor population. Loss of Wnt signaling does not appear to lead to their death, although the extremely efficient scavenger mechanisms active in the thymus make quantification of apoptotic cells difficult in the thymus. These results also provide further evidence to support the previously identified link between Wnt signaling in TECs and regulation of Foxn1 expression [38] and further suggest that the thymic degeneration observed in response to Wnt signaling inhibition may be mediated by a reduction in Foxn1 dosage. Thymic involution and the subsequent loss of capacity to generate sufficient functional T cells represents a central aspect of the ageing immune system which at least in part contributes to an increased susceptibility to infection, development of autoimmune diseases, and cancer in the aging population. Future studies should address the downstream targets of Wnt signaling, which are responsible for maintenance of TEC progenitors and thymic epithelial microenvironments as they may provide useful targets for therapies aimed at counteracting age associated thymic involution or the premature thymic degeneration associated with cancer therapy and bone marrow transplants.

## Supporting Information

Figure S1 Decreased Foxn1 protein expression in both cTECs and mTECs in response to DKK1. Immunofluorescent staining of thymic sections derived from 4 week Dox fed TetO-Dkk1 transgenic mice revealed a dramatic decrease in FoxN1 protein expression (green nuclei) within both DEC205+ cortical areas (red) and K14^+^ medullary areas (blue) (A–D) when compared with identically treated K5rtTA-ST littermate controls. (E–H) Magnification = 200×. Scale bars = 100 µm.
(2.57 MB TIF)

Figure S2 Increased K5K8DP TECs in response to Wnt stimulation in vivo and in vitro. Immunofluorescent staining of thymic sections derived from 6-week-old K14Wnt7a transgenic mice revealed an abundance of K5K8DP potential TEC progenitors scattered throughout the thymus including most of the cortex (A–D), while littermate control mice showed a normal distribution of K5+TECs dominating the medulla with most K5K8DP TECs localized to the cortico-medullary junction (F–I). Visualization of the cortico-medullary junction at 400X revealed a less defined boundary and abundant K5K8DP TECs in the K14Wnt7a mice with almost no K8SP mature mTECs (E). In contrast, thymic sections from control mice had a defined cortico-medullary junction, fewer K5K8DP TECs and abundant K8SP mature mTECs (J, white arrows show K8SP mTECs). A similar abundance of K5K8DP TECs and loss of defined cortical and medullary areas was observed in sections derived from E15.5 FTOCs following 72 hrs of culture in Wnt3a conditioned medium (K, upper row). Littermate E15.5 FTOCs cultured in FTOC medium in the absence of Wnt3a exhibited fewer K5K8DP TECs and more defined cortical and medullary areas (K, lower row).
(4.90 MB TIF)

## References

[1] Anderson G, Jenkinson EJ (2001). Lymphostromal interactions in thymic development and function.. Nat Rev Immunol.

[2] Savage PA, Davis MM (2001). A kinetic window constricts the T cell receptor repertoire in the
thymus.. Immunity.

[3] van Ewijk W, Wang B, Hollander G, Kawamoto H, Spanopoulou E (1999). Thymic microenvironments, 3-D versus 2-D?. Semin Immunol.

[4] van Ewijk W, Hollander G, Terhorst C, Wang B (2000). Stepwise development of thymic microenvironments in vivo is
regulated by thymocyte subsets.. Development.

[5] Itoi M, Tsukamoto N, Yoshida H, Amagai T (2007). Mesenchymal cells are required for functional development of
thymic epithelial cells.. Int Immunol.

[6] Jenkinson WE, Rossi SW, Parnell SM, Jenkinson EJ, Anderson G (2007). PDGFRalpha-expressing mesenchyme regulates thymus growth and the
availability of intrathymic niches.. Blood.

[7] Rossi SW, Kim MY, Leibbrandt A, Parnell SM, Jenkinson WE (2007). RANK signals from CD4+3− inducer cells
regulate development of Aire-expressing epithelial cells in the thymic
medulla.. J Exp Med.

[8] Shores EW, Van Ewijk W, Singer A (1991). Disorganization and restoration of thymic medullary epithelial
cells in T cell receptor-negative scid mice: evidence that receptor-bearing
lymphocytes influence maturation of the thymic microenvironment.. Eur J Immunol.

[9] Surh CD, Ernst B, Sprent J (1992). Growth of epithelial cells in the thymic medulla is under the
control of mature T cells.. J Exp Med.

[10] Akiyama T, Shimo Y, Yanai H, Qin J, Ohshima D (2008). The tumor necrosis factor family receptors RANK and CD40
cooperatively establish the thymic medullary microenvironment and
self-tolerance.. Immunity.

[11] Hikosaka Y, Nitta T, Ohigashi I, Yano K, Ishimaru N (2008). The cytokine RANKL produced by positively selected thymocytes
fosters medullary thymic epithelial cells that express autoimmune regulator.. Immunity.

[12] Irla M, Hugues S, Gill J, Nitta T, Hikosaka Y (2008). Autoantigen-specific interactions with CD4+ thymocytes
control mature medullary thymic epithelial cell cellularity.. Immunity.

[13] Ferrick DA, Sambhara SR, Ballhausen W, Iwamoto A, Pircher H (1989). T cell function and expression are dramatically altered in T cell
receptor V gamma 1.1J gamma 4C gamma 4 transgenic mice.. Cell.

[14] Barclay AN, Mayrhofer G (1981). Bone marrow origin of Ia-positive cells in the medulla rat
thymus.. J Exp Med.

[15] Gotter J, Brors B, Hergenhahn M, Kyewski B (2004). Medullary epithelial cells of the human thymus express a highly
diverse selection of tissue-specific genes colocalized in chromosomal
clusters.. J Exp Med.

[16] Kyewski B, Derbinski J (2004). Self-representation in the thymus: an extended view.. Nat Rev Immunol.

[17] Gillard GO, Farr AG (2006). Features of medullary thymic epithelium implicate postnatal
development in maintaining epithelial heterogeneity and tissue-restricted
antigen expression.. J Immunol.

[18] Anderson MS, Venanzi ES, Klein L, Chen Z, Berzins SP (2002). Projection of an immunological self shadow within the thymus by
the aire protein.. Science.

[19] Gabor MJ, Godfrey DI, Scollay R (1997). Recent thymic emigrants are distinct from most medullary
thymocytes.. Eur J Immunol.

[20] Clevers H (2006). Wnt/beta-catenin signaling in development and disease.. Cell.

[21] Scheller M, Huelsken J, Rosenbauer F, Taketo MM, Birchmeier W (2006). Hematopoietic stem cell and multilineage defects generated by
constitutive beta-catenin activation.. Nat Immunol.

[22] Staal FJ, Luis TC, Tiemessen MM (2008). WNT signalling in the immune system: WNT is spreading its wings.. Nat Rev Immunol.

[23] Fleming HE, Janzen V, Lo Celso C, Guo J, Leahy KM (2008). Wnt signaling in the niche enforces hematopoietic stem cell
quiescence and is necessary to preserve self-renewal in vivo.. Cell Stem Cell.

[24] Korinek V, Barker N, Moerer P, van Donselaar E, Huls G (1998). Depletion of epithelial stem-cell compartments in the small
intestine of mice lacking Tcf-4.. Nat Genet.

[25] Miller JR (2002). The Wnts.. Genome Biol.

[26] Niehrs C (1999). Head in the WNT: the molecular nature of Spemann's head
organizer.. Trends Genet.

[27] Leyns L, Bouwmeester T, Kim SH, Piccolo S, De Robertis EM (1997). Frzb-1 is a secreted antagonist of Wnt signaling expressed in the
Spemann organizer.. Cell.

[28] Wodarz A, Nusse R (1998). Mechanisms of Wnt signaling in development.. Annu Rev Cell Dev Biol.

[29] Wu W, Glinka A, Delius H, Niehrs C (2000). Mutual antagonism between dickkopf1 and dickkopf2 regulates
Wnt/beta-catenin signalling.. Curr Biol.

[30] Glinka A, Wu W, Delius H, Monaghan AP, Blumenstock C (1998). Dickkopf-1 is a member of a new family of secreted proteins and
functions in head induction.. Nature.

[31] Mukhopadhyay M, Shtrom S, Rodriguez-Esteban C, Chen L, Tsukui T (2001). Dickkopf1 is required for embryonic head induction and limb
morphogenesis in the mouse.. Dev Cell.

[32] Grotewold L, Ruther U (2002). Bmp, Fgf and Wnt signalling in programmed cell death and
chondrogenesis during vertebrate limb development: the role of Dickkopf-1.. Int J Dev Biol.

[33] Grotewold L, Ruther U (2002). The Wnt antagonist Dickkopf-1 is regulated by Bmp signaling and
c-Jun and modulates programmed cell death.. Embo J.

[34] Huelsken J, Vogel R, Erdmann B, Cotsarelis G, Birchmeier W (2001). beta-Catenin controls hair follicle morphogenesis and stem cell
differentiation in the skin.. Cell.

[35] Shu W, Guttentag S, Wang Z, Andl T, Ballard P (2005). Wnt/beta-catenin signaling acts upstream of N-myc, BMP4, and FGF
signaling to regulate proximal-distal patterning in the lung.. Dev Biol.

[36] Liu F, Thirumangalathu S, Gallant NM, Yang SH, Stoick-Cooper CL (2007). Wnt-beta-catenin signaling initiates taste papilla development.. Nat Genet.

[37] Ito M, Yang Z, Andl T, Cui C, Kim N (2007). Wnt-dependent de novo hair follicle regeneration in adult mouse
skin after wounding.. Nature.

[38] Balciunaite G, Keller MP, Balciunaite E, Piali L, Zuklys S (2002). Wnt glycoproteins regulate the expression of FoxN1, the gene
defective in nude mice.. Nat Immunol.

[39] Osada M, Ito E, Vazquez-Cintron E, Venkatesh T, Friedel RH (2006). The Wnt Signaling Antagonist Kremen1 is Required for the
Development of Thymic Architecture.. Clinical and Developmental Immunology.

[40] Pongracz J, Hare K, Harman B, Anderson G, Jenkinson EJ (2003). Thymic epithelial cells provide WNT signals to developing
thymocytes.. Eur J Immunol.

[41] Weerkamp F, Baert MR, Naber BA, Koster EE, de Haas EF (2006). Wnt signaling in the thymus is regulated by differential
expression of intracellular signaling molecules.. Proc Natl Acad Sci U S A.

[42] Hattori N, Kawamoto H, Katsura Y (1996). Isolation of the most immature population of murine fetal
thymocytes that includes progenitors capable of generating T, B, and myeloid
cells.. J Exp Med.

[43] Hattori N, Kawamoto H, Fujimoto S, Kuno K, Katsura Y (1996). Involvement of transcription factors TCF-1 and GATA-3 in the
initiation of the earliest step of T cell development in the thymus.. J Exp Med.

[44] Ioannidis V, Beermann F, Clevers H, Held W (2001). The beta-catenin–TCF-1 pathway ensures
CD4(+)CD8(+) thymocyte survival.. Nat Immunol.

[45] Schilham MW, Wilson A, Moerer P, Benaissa-Trouw BJ, Cumano A (1998). Critical involvement of Tcf-1 in expansion of thymocytes.. J Immunol.

[46] Staal FJ, Meeldijk J, Moerer P, Jay P, van de Weerdt BC (2001). Wnt signaling is required for thymocyte development and activates
Tcf-1 mediated transcription.. Eur J Immunol.

[47] Kuraguchi M, Wang XP, Bronson RT, Rothenberg R, Ohene-Baah NY (2006). Adenomatous polyposis coli (APC) is required for normal
development of skin and thymus.. PLoS Genet.

[48] Reya T, Duncan AW, Ailles L, Domen J, Scherer DC (2003). A role for Wnt signalling in self-renewal of haematopoietic stem
cells.. Nature.

[49] Zuklys S, Gill J, Keller MP, Hauri-Hohl M, Zhanybekova S (2009). Stabilized beta-catenin in thymic epithelial cells blocks thymus
development and function.. J Immunol.

[50] Chu EY, Hens J, Andl T, Kairo A, Yamaguchi TP (2004). Canonical WNT signaling promotes mammary placode development and
is essential for initiation of mammary gland morphogenesis.. Development.

[51] Diamond I, Owolabi T, Marco M, Lam C, Glick A (2000). Conditional gene expression in the epidermis of transgenic mice
using the tetracycline-regulated transactivators tTA and rTA linked to the
keratin 5 promoter.. J Invest Dermatol.

[52] Zhang Y, Andl T, Yang SH, Teta M, Liu F (2008). Activation of beta-catenin signaling programs embryonic epidermis
to hair follicle fate.. Development.

[53] Andl T, Reddy ST, Gaddapara T, Millar SE (2002). WNT signals are required for the initiation of hair follicle
development.. Dev Cell.

[54] van de Wetering M, de Lau W, Clevers H (2002). WNT signaling and lymphocyte development.. Cell.

[55] Klug DB, Carter C, Gimenez-Conti IB, Richie ER (2002). Cutting edge: thymocyte-independent and thymocyte-dependent
phases of epithelial patterning in the fetal thymus.. J Immunol.

[56] Klug DB, Carter C, Crouch E, Roop D, Conti CJ (1998). Interdependence of cortical thymic epithelial cell
differentiation and T-lineage commitment.. Proc Natl Acad Sci U S A.

[57] Aw D, Silva AB, Maddick M, von Zglinicki T, Palmer DB (2008). Architectural changes in the thymus of aging mice.. Aging Cell.

[58] Takeoka Y, Chen SY, Yago H, Boyd R, Suehiro S (1996). The murine thymic microenvironment: changes with age.. Int Arch Allergy Immunol.

[59] Candi E, Rufini A, Terrinoni A, Giamboi-Miraglia A, Lena AM (2007). DeltaNp63 regulates thymic development through enhanced
expression of FgfR2 and Jag2.. Proc Natl Acad Sci U S A.

[60] Senoo M, Pinto F, Crum CP, McKeon F (2007). p63 Is essential for the proliferative potential of stem cells in
stratified epithelia.. Cell.

[61] Mills AA, Zheng B, Wang XJ, Vogel H, Roop DR (1999). p63 is a p53 homologue required for limb and epidermal
morphogenesis.. Nature.

[62] Parsa R, Yang A, McKeon F, Green H (1999). Association of p63 with proliferative potential in normal and
neoplastic human keratinocytes.. J Invest Dermatol.

[63] Yang A, Schweitzer R, Sun D, Kaghad M, Walker N (1999). p63 is essential for regenerative proliferation in limb,
craniofacial and epithelial development.. Nature.

[64] Lee H, Kimelman D (2002). A dominant-negative form of p63 is required for epidermal
proliferation in zebrafish.. Dev Cell.

[65] Truong AB, Kretz M, Ridky TW, Kimmel R, Khavari PA (2006). p63 regulates proliferation and differentiation of
developmentally mature keratinocytes.. Genes Dev.

[66] Kuhnert F, Davis CR, Wang HT, Chu P, Lee M (2004). Essential requirement for Wnt signaling in proliferation of adult
small intestine and colon revealed by adenoviral expression of Dickkopf-1.. Proc Natl Acad Sci U S A.

[67] Luis TC, Weerkamp F, Naber BA, Baert MR, de Haas EF (2009). Wnt3a deficiency irreversibly impairs hematopoietic stem cell
self-renewal and leads to defects in progenitor cell differentiation.. Blood.

[68] Pinto D, Gregorieff A, Begthel H, Clevers H (2003). Canonical Wnt signals are essential for homeostasis of the
intestinal epithelium.. Genes Dev.

[69] Mulroy T, McMahon JA, Burakoff SJ, McMahon AP, Sen J (2002). Wnt-1 and Wnt-4 regulate thymic cellularity.. Eur J Immunol.

[70] Mulroy T, Xu Y, Sen JM (2003). beta-Catenin expression enhances generation of mature thymocytes.. Int Immunol.

[71] Heng TS, Goldberg GL, Gray DH, Sutherland JS, Chidgey AP (2005). Effects of castration on thymocyte development in two different
models of thymic involution.. J Immunol.

[72] Hirokawa K, Utsuyama M, Kasai M, Kurashima C, Ishijima S (1994). Understanding the mechanism of the age-change of thymic function
to promote T cell differentiation.. Immunol Lett.

[73] Utsuyama M, Hirokawa K, Mancini C, Brunelli R, Leter G (1995). Differential effects of gonadectomy on thymic stromal cells in
promoting T cell differentiation in mice.. Mech Ageing Dev.

[74] Kwack MH, Sung YK, Chung EJ, Im SU, Ahn JS (2008). Dihydrotestosterone-inducible dickkopf 1 from balding dermal
papilla cells causes apoptosis in follicular keratinocytes.. J Invest Dermatol.

[75] Min D, Panoskaltsis-Mortari A, Kuro OM, Hollander GA, Blazar BR (2007). Sustained thymopoiesis and improvement in functional immunity
induced by exogenous KGF administration in murine models of aging.. Blood.

[76] Flores KG, Li J, Sempowski GD, Haynes BF, Hale LP (1999). Analysis of the human thymic perivascular space during aging.. J Clin Invest.

[77] Gray DH, Seach N, Ueno T, Milton MK, Liston A (2006). Developmental kinetics, turnover, and stimulatory capacity of
thymic epithelial cells.. Blood.

[78] Rossi SW, Jenkinson WE, Anderson G, Jenkinson EJ (2006). Clonal analysis reveals a common progenitor for thymic cortical
and medullary epithelium.. Nature.

[79] Dooley J, Erickson M, Farr AG (2008). Alterations of the medullary epithelial compartment in the
Aire-deficient thymus: implications for programs of thymic epithelial
differentiation.. J Immunol.

[80] Gillard GO, Dooley J, Erickson M, Peltonen L, Farr AG (2007). Aire-dependent alterations in medullary thymic epithelium
indicate a role for Aire in thymic epithelial differentiation.. J Immunol.

[81] Gray D, Abramson J, Benoist C, Mathis D (2007). Proliferative arrest and rapid turnover of thymic epithelial
cells expressing Aire.. J Exp Med.

[82] Laufer TM, DeKoning J, Markowitz JS, Lo D, Glimcher LH (1996). Unopposed positive selection and autoreactivity in mice
expressing class II MHC only on thymic cortex.. Nature.

[83] Liston A, Nutsch KM, Farr AG, Lund JM, Rasmussen JP (2008). Differentiation of regulatory Foxp3+ T cells in the
thymic cortex.. Proc Natl Acad Sci U S A.

[84] Pellegrini G, Dellambra E, Golisano O, Martinelli E, Fantozzi I (2001). p63 identifies keratinocyte stem cells.. Proc Natl Acad Sci U S A.

[85] Chilosi M, Zamo A, Brighenti A, Malpeli G, Montagna L (2003). Constitutive expression of DeltaN-p63alpha isoform in human
thymus and thymic epithelial tumours.. Virchows Arch.

[86] Dotto J, Pelosi G, Rosai J (2007). Expression of p63 in thymomas and normal thymus.. Am J Clin Pathol.

[87] Irifune T, Tamechika M, Adachi Y, Tokuda N, Sawada T (2004). Morphological and immunohistochemical changes to thymic
epithelial cells in the irradiated and recovering rat thymus.. Arch Histol Cytol.

[88] Kikuchi T, Ichimiya S, Kojima T, Crisa L, Koshiba S (2004). Expression profiles and functional implications of p53-like
transcription factors in thymic epithelial cell subtypes.. Int Immunol.

[89] Lena AM, Shalom-Feuerstein R, Rivetti di Val Cervo P, Aberdam D, Knight RA (2008). miR-203 represses ‘stemness’ by repressing
DeltaNp63.. Cell Death Differ.

[90] Yi R, Poy MN, Stoffel M, Fuchs E (2008). A skin microRNA promotes differentiation by repressing
‘stemness’.. Nature.

[91] Shakib S, Desanti GE, Jenkinson WE, Parnell SM, Jenkinson EJ (2009). Checkpoints in the development of thymic cortical epithelial
cells.. J Immunol.

[92] Blanpain C, Fuchs E (2009). Epidermal homeostasis: a balancing act of stem cells in the skin.. Nat Rev Mol Cell Biol.

[93] Baba Y, Yokota T, Spits H, Garrett KP, Hayashi S (2006). Constitutively active beta-catenin promotes expansion of
multipotent hematopoietic progenitors in culture.. J Immunol.

