# Supplementary figures and images for: DKK1 Mediated Inhibition of Wnt Signaling in Postnatal Mice Leads to Loss of TEC Progenitors and Thymic Degeneration
Source: PLoS One. 2010 Feb 8;5(2):e9062. doi: 10.1371/journal.pone.0009062 (PMC2817005; doi:10.1371/journal.pone.0009062)

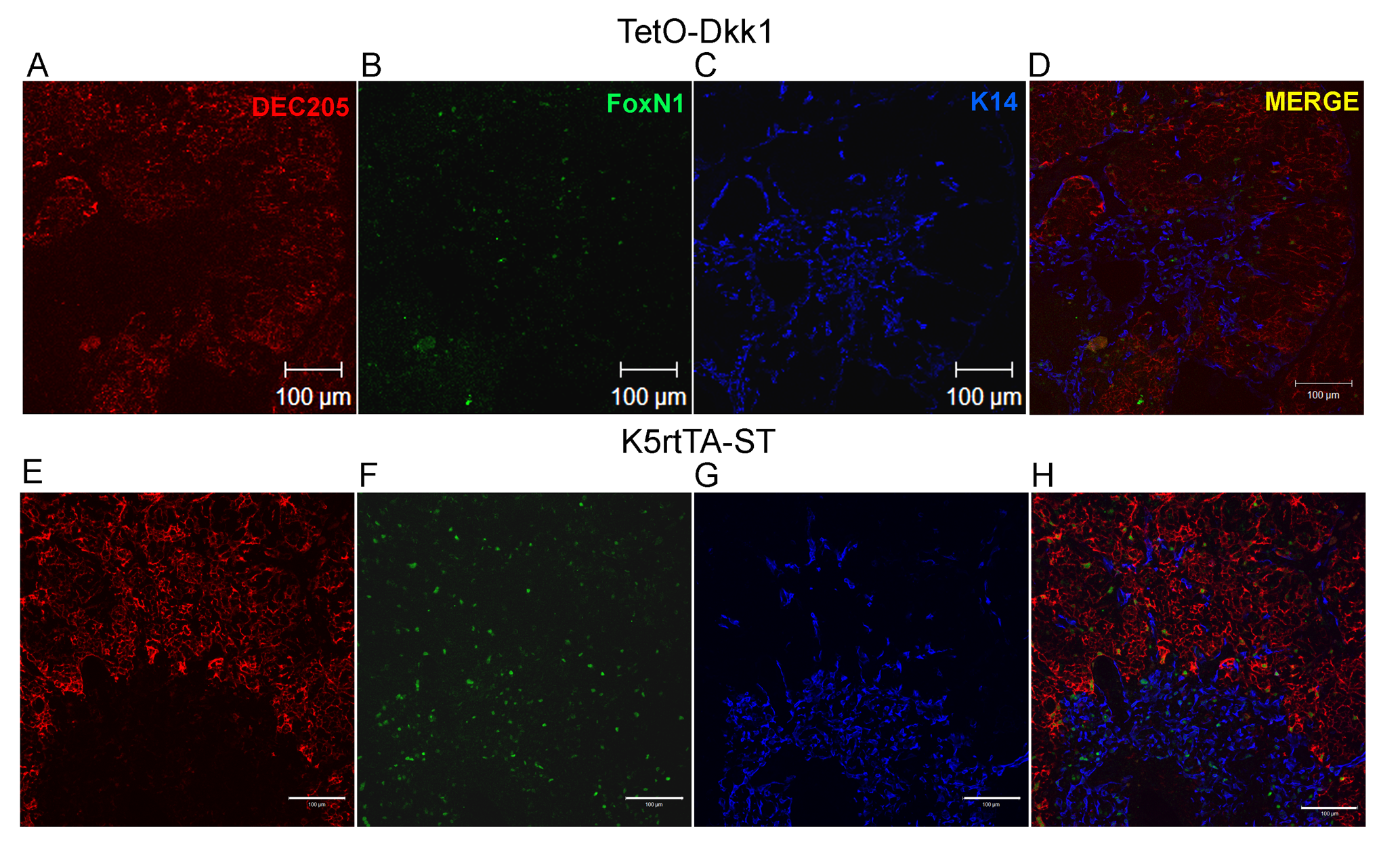

Supplement: Figure S1 — Decreased Foxn1 protein expression in both cTECs and mTECs in response to DKK1. Immunofluorescent staining of thymic sections derived from 4 week Dox fed TetO-Dkk1 transgenic mice revealed a dramatic decrease in FoxN1 protein expression (green nuclei) within both DEC205+ cortical areas (red) and K14+ medullary areas (blue) (A–D) when compared with identically treated K5rtTA-ST littermate controls. (E–H) Magnification = 200×. Scale bars = 100 µm. (2.57 MB TIF) [file pone.0009062.s001.tif]

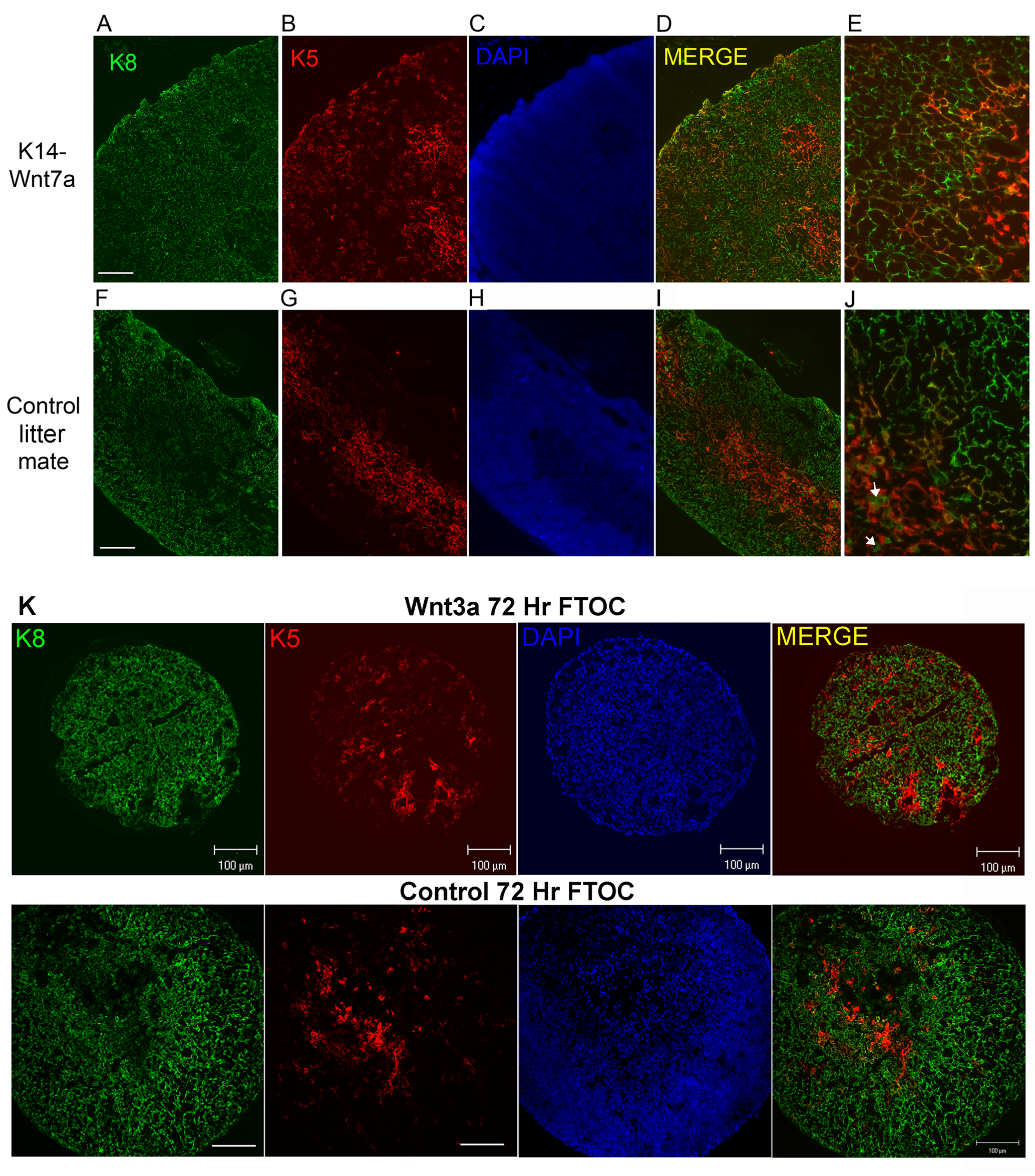

Supplement: Figure S2 — Increased K5K8DP TECs in response to Wnt stimulation in vivo and in vitro. Immunofluorescent staining of thymic sections derived from 6-week-old K14Wnt7a transgenic mice revealed an abundance of K5K8DP potential TEC progenitors scattered throughout the thymus including most of the cortex (A–D), while littermate control mice showed a normal distribution of K5+TECs dominating the medulla with most K5K8DP TECs localized to the cortico-medullary junction (F–I). Visualization of the cortico-medullary junction at 400X revealed a less defined boundary and abundant K5K8DP TECs in the K14Wnt7a mice with almost no K8SP mature mTECs (E). In contrast, thymic sections from control mice had a defined cortico-medullary junction, fewer K5K8DP TECs and abundant K8SP mature mTECs (J, white arrows show K8SP mTECs). A similar abundance of K5K8DP TECs and loss of defined cortical and medullary areas was observed in sections derived from E15.5 FTOCs following 72 hrs of culture in Wnt3a conditioned medium (K, upper row). Littermate E15.5 FTOCs cultured in FTOC medium in the absence of Wnt3a exhibited fewer K5K8DP TECs and more defined cortical and medullary areas (K, lower row). (4.90 MB TIF) [file pone.0009062.s002.tif]
